# Charge‐Compensated N‐Doped *π*‐Conjugated Polymers: Toward both Thermodynamic Stability of N‐Doped States in Water and High Electron Conductivity

**DOI:** 10.1002/advs.202203530

**Published:** 2022-09-05

**Authors:** Fabian Borrmann, Takuya Tsuda, Olga Guskova, Nataliya Kiriy, Cedric Hoffmann, David Neusser, Sabine Ludwigs, Uwe Lappan, Frank Simon, Martin Geisler, Bipasha Debnath, Yulia Krupskaya, Mahmoud Al‐Hussein, Anton Kiriy

**Affiliations:** ^1^ Leibniz‐Institut für Polymerforschung Dresden e.V Hohe Straße 6 01069 Dresden Germany; ^2^ Dresden Center for Computational Materials Science (DCMS) TU Dresden 01062 Dresden Germany; ^3^ IPOC‐Functional Polymers Institute of Polymer Chemistry & Center for Integrated Quantum Science and Technology (IQST) University of Stuttgart Pfaffenwaldring 55 70569 Stuttgart Germany; ^4^ Leibniz‐Institut für Festkörper‐ und Werkstoffforschung Dresden Helmholtzstraße 20 01069 Dresden Germany; ^5^ Physics Department and Hamdi Mango Center for Scientific Research The University of Jordan Amman 11942 Jordan

**Keywords:** density functional theory calculations, electron conductivity, n‐doped states, thermodynamic stability, *π*‐conjugated polymers

## Abstract

The understanding and applications of electron‐conducting *π*‐conjugated polymers with naphtalene diimide (NDI) blocks show remarkable progress in recent years. Such polymers demonstrate a facilitated n‐doping due to the strong electron deficiency of the main polymer chain and the presence of the positively charged side groups stabilizing a negative charge of the n‐doped backbone. Here, the n‐type conducting NDI polymer with enhanced stability of its n‐doped states for prospective “in‐water” applications is developed. A combined experimental–theoretical approach is used to identify critical features and parameters that control the doping and electron transport process. The facilitated polymer reduction ability and the thermodynamic stability in water are confirmed by electrochemical measurements and doping studies. This material also demonstrates a high conductivity of 10^−2^ S cm^−1^ under ambient conditions and 10^−1^ S cm^−1^ in vacuum. The modeling explains the stabilizing effects  for various dopants. The simulations show a significant doping‐induced “collapse” of the positively charged side chains on the core bearing a partial negative charge. This explains a decrease in the lamellar spacing observed in experiments. This study fundamentally enables a novel pathway for achieving both thermodynamic stability of the n‐doped states in water and the high electron conductivity of polymers.

## Graphical abstract

This work focuses on electron‐conducting *π*‐conjugated polymers with naphtalene diimide blocks bearing positively charged side groups. Studied polymers are characterized by enhanced stability of their n‐doped states for prospective “in‐water” applications and high election conductivity 10^−2^ S cm^−1^ under ambient conditions. Using a combination of experimental and theoretical approaches, the authors explain the doping‐induced processes in thin polymer films.

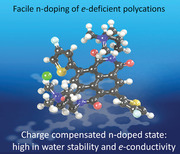

## 1 Introduction

Organic semiconductors (OSCs) and, particularly, *π*‐conjugated polymers are receiving increasing attention due to their valuable optoelectronic and mechanical properties, and the possibility for low‐cost production.^[^ ^1^ ^]^ During the last two decades, researchers’ efforts were mostly directed toward the development of materials for novel devices such as organic transistors, organic light‐emitting diodes, and solar cells^[^ ^1^, ^2^, ^3^ ^]^ and some of the achievements in these fields are already commercialized.^[^ ^4^ ^]^ Nowadays, emerging fields known as artificial intelligence and the internet of things,^[^ ^5^ ^]^ healthcare,^[^ ^6^ ^]^ and bioelectronics,^[^ ^7^ ^]^ undergo rapid development. These fields require new devices, such as diverse chemo‐ and bio‐sensors,^[^ ^8^ ^]^ photodetectors,^[^ ^9^, ^10^ ^]^ thermoelectric devices,^[^ ^11^ ^]^ electrochemical transistors,^[^ ^12^ ^]^ and neuromorphic and synaptic devices.^[^ ^13^, ^14^ ^]^ Many of these devices operate in an open atmosphere and in a water environment.^[^ ^15^ ^]^ Because of their excellent environmental and operational stability and attractive electronic characteristics, poly(3,4‐ethylenedioxythiophene): poly(styrenesulfonate) (PEDOT:PSS)^[^ ^16^ ^]^ and a few other polymers, such as polypyrrole and polyaniline, are the most common polymeric materials used for these applications.^[^ ^12^, ^13^, ^14^, ^15^, ^16^, ^17^, ^18^ ^]^ While all these polymers are hole‐conductors, n‐type conductors are of great importance, as they would allow the fabrication of complementary logic circuits and a dramatic sophistication of bioelectronics devices, such as complex iontronic delivery systems, surface switch devices, diverse metabolite detection devices, and so on.^[^ ^19^, ^20^, ^21^, ^22^ ^]^ However, the implementation of electron conductivity in OSCs is a difficult task because electrons are transported via semiconductors’ LUMOs (lowest unoccupied molecular orbitals), the energy of which for most of the OSCs lies well above the reduction potentials of environmental oxidants – water and oxygen. To define the electronic characteristics of OSCs suitable for the conduction of electrons in the presence of water and/or oxygen, redox reactions responsible for the oxidative degradation of the n‐doped states must be identified. In general, pH‐dependent reduction potentials of water and oxygen lie in the ranges of −3.6 to −4.44 eV and −4.8 to −5.67 eV,^[^ ^23^ ^]^ respectively, which would dramatically restrict the range of environmentally stable electron conductors. Fortunately, these redox reactions require proper catalysts in order to take place whereas, under catalyst‐free conditions, they are going not necessarily fast enough with typical OSCs.^[^ ^24^, ^25^ ^]^ Brédas et al. identified a potential of −3.6 eV as a threshold for OSCs’ LUMO, which defines the environmental stability of the corresponding n‐doped states.^[^ ^26^ ^]^ It was shown for a few OSCs with a LUMO energy above −3.6 eV that they undergo a rapid degradation of the n‐doped states, whereas OSCs with a LUMO below −3.6 eV exhibit environmental stability which allowed their patterning at ambient conditions.^[^ ^27^ ^]^ The threshold was attributed to the oxidative reactions of O_2_(H_2_O)_2_ complexes having the reduction potential of −3.6 eV. At the same time, other works demonstrated that OSCs having LUMO energies in the −3.8 eV to −4.1 eV range, that is, those which formally fulfill the above‐formulated stability rule, still strongly suffer from environmental water and oxygen.^[^ ^28^, ^29^ ^]^ Obviously, the operation of n‐type OSCs in devices poses higher requirements to the stability than their handling during the device fabrication process.

To circumvent the environmental stability issue of electron‐conducting polymers, various electron‐deficient groups have been incorporated into the polymer repeat unit. For example, a strongly electron‐deficient naphthalenediimine (NDI) is a popular building block for designing n‐type semiconductors and NDI‐bithiophene copolymer P(NDIOD‐T2) with its LUMO of ‐4.1 eV was a benchmark semiconductor for n‐type OFETs for a long time.^[^ ^30^, ^31^, ^32^, ^33^ ^]^ Although P(NDIOD‐T2) shows a good performance in n‐type transistors, the n‐doped P(NDIOD‐T2) is still unstable in water and it shows a conductivity of 10^–3^ S cm^–1^ only under inert conditions.^[^ ^34^, ^35^, ^36^, ^37^, ^38^ ^]^ The replacement of the thiophene rings in P(NDIOD‐T2) by thiadiazoles further shifts the LUMO down by 0.2 eV, which improves the n‐doping ability and increases conductivity up to 10^–1^ S cm^–1^. However, the polymer is still unstable in the n‐doped state in the presence of water.^[^ ^39^ ^]^

A copolymerization of two super‐strong electron‐accepting groups naphtho[2,3‐b:6,7‐b″]dithiophenediimide (NDTI) and benzobisthiadiazole (benzo[1,2‐c:4,5‐c″]‐bis[1,2,5]thiadiazole (BBT)) units gave rise to NDTI–BBT copolymer with electron conductivity of 5 S cm^–1^ and a LUMO of ‐4.4 eV. To the best of our knowledge, this is one of the lowest LUMO reported in literature among all polymers. Given this low LUMO value, the copolymer could have certain water stability in the n‐doped state, which has not been reported yet.^[^ ^40^ ^]^

Other electron‐deficient building blocks were employed to produce n‐conductors with enhanced stability.^[^ ^41^ ^]^ Katz and coworkers have introduced chlorinated and fluorinated copolymers comprising electron‐deficient unit benzodifurandione‐based oligo(*p*‐phenylene vinylene) (BDOPV) and the weak donor moiety dichlorodithienylethene (ClTVT).^[^ ^42^ ^]^ The produced copolymers possessed low‐lying LUMO of −4.3 eV and exhibited high electron conductivities in the N‐DMBI and cobaltocene CoCp_2_‐doped state (above 1 S cm^−1^) and excellent air stability over hundreds of days. Enhanced environmental stability of the n‐doped state of BDOPV–ClTVT copolymer was reported,^[^ ^42^, ^43^ ^]^ which was attributed to a self‐encapsulation of the polymer phase due to a high polymer crystallinity and close packing of the hydrophobic side chains, preventing the penetration of air and moisture. The incorporation of kinks into the main chains^[^ ^44^ ^]^ and modifications of the side chains^[^ ^45^ ^]^ to increase the miscibility of polymers with dopants were applied for improving n‐doping ability. A conductivity of about 1 S cm^−1^ was reported for triethylene glycol‐substituted polymer PNDI2TED‐2Tz, however, even in this case, the n‐doped PNDI2TED‐2Tz exhibited low environmental stability.^[^ ^45^ ^]^

Another method to reduce LUMO assumes a rigidification of the conjugated backbone to stabilize the planar conformation. Together with the incorporation of strongly withdrawing groups, this approach allows one to push the LUMO down to −4.49 eV and achieve good environmental stability of the polymer in its n‐doped state. To the best of our knowledge, this ladder polymer and the NDTI‐BBT‐based polymer reported by Takimiya et al.^[^ ^40^ ^]^ possess the lowest LUMOs reported in literature among all polymers.

Casado et al. have introduced an interesting approach for placing the LUMO energy level below −4.6 eV in conjugated oligomers, which benefit from the presence of strongly electron‐withdrawing dicyano‐methylene groups and a possibility for quinoidal‐to‐aromatic diradical conversion. These oligomers possess high stability in the n‐doping state and electron conductivity of 14.0 S cm^−1^.^[^ ^46^ ^]^

An interesting class of polymers comprising *π*‐conjugated backbones and cationic side groups, named self‐doped polymers, was introduced more than 30 years ago by Wudl^[^ ^47^ ^]^ and further developed by Cao et al.^[^ ^48^ ^]^ The latter polymers were applied as cathode interlayer materials which improve the efficiency of solar cells and OLEDs, through improving electron‐injection and ‐collection processes.^[^ ^49^, ^50^ ^]^ The early‐reported n‐type self‐doped polymers contained electron‐rich backbones (polyacetylene, polyfluorene, etc.) so achieving truly stable n‐doped states was hardly possible even with the incorporation of the cationic side chains.

Recently, conjugated polycations (CPCs) containing electron‐deficient naphthalene diimide‐based main chains were identified as the most efficient cathode interlayers.^[^ ^51^, ^52^, ^53^, ^54^ ^]^ The improved electron‐injection efficiency of the NDI‐based polycations was ascribed to a facilitated n‐doping, which in turn, was attributed to a combination of two structural factors – the strong electron‐deficiency of the main chain and presence of positively charged side groups stabilizing the negative charge of the n‐doped backbone.^[^ ^55^ ^]^ According to Tang et al.,^[^ ^55^ ^]^ the highest stabilization of the n‐doped state is observed in a so‐called, self‐compensated doped state, formed after the release of the intrinsic anions (anions, which were present in NDI‐CPCs before doping). Whereas charge injection applications of the NDI‐based polycations are well‐reported, the doping phenomenon and the role of ionic interactions on the doping and the charge transport processes have received less attention.

**Figure 1:**
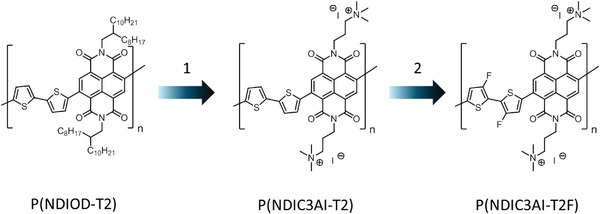
Polymers studied in this work: compared to P(NDIOD‐T2) and P(NDIC3AI‐T2), P(NDIC3AI‐T2F), represents a two‐fold stabilization of the n‐doped state – by adding the 1) cationic side chains and incorporation of the 2) electron‐deficient fluorine atoms.

The main objective of the present work is the development of n‐type conducting materials with enhanced stability for prospective “in‐water” applications. To this end, two NDI‐based *π*‐conjugated polymers, P(NDIC3AI‐T2) and P(NDIC3AI‐T2F) (**Figure** **1**) having cationic side groups have been prepared and investigated in detail by a combination of experimental and theoretical methods. We focused on understanding the role of ionic groups in the doping and electron transport processes.

We show that the n‐doped state of a prototypical n‐type polymer P(NDIOD‐T2), having a LUMO in the −3.8 to −4.1 eV range, degrades within minutes in the air and the n‐doped state cannot be restored in vacuum or by heat treatments. This suggests that the above‐mentioned energy threshold of −3.8 eV^[^ ^26^, ^27^ ^]^ for OSC's LUMOs describes the ability of n‐doped OSCs to maintain stability against oxidation only during short‐term exposure to air. Obviously, more electropositive oxidative reactions than reactions of O_2_(H_2_O)_2_ take place relatively fast in the n‐doped films. We suggest that for achieving truly thermodynamically stable n‐doped states, at least against water, it is essential to place the LUMO of the OSCs below the range of the pH‐dependent reduction potential of water, that is, below −4.44 eV versus vacuum.^[^ ^56^ ^]^

We demonstrate that our newly synthesized polymer comprising a high electron‐deficiency moiety in the main chain and stabilizing cationic side groups has a decent fraction of reduced states in the potential below −4.5 eV, leading to the long‐term stability of its n‐doped state in the water solution state. However, the polymer degrades upon long storage in air, expectedly showing that oxygen is a more severe oxidative hazard. This problem could be partly circumvented by excessive doping of the cationic polymers with oxalate anions, which not only cause immediate n‐doping but also act as latent dopants, due to the accumulation of their excessive amount in the polymer film due to the ionic interactions.

## 2 Results and Discussion

### 2.1 Synthesis

To evaluate the influence of cationic side groups on the n‐doping ability of *π*‐conjugated polymers, a previously reported polymer P(NDIC3AI‐T2) having electron‐deficient NDI‐dithiophene main chain and trimethyl‐propyl‐ammonium side groups, was synthesized (Figure 1).^[^ ^55^ ^]^ A new fluorinated polymer P(NDIC3AI‐T2F) was also synthesized, which allowed to exploit the combined effect of the cationic side groups and enhanced electron deficiency provided by the electron‐withdrawing fluorine atoms. The third polymer, P(NDIOD‐T2), which has neither ionic side groups nor fluorine atoms, was chosen as a reference. The greatest difficulty in the synthesis of the ionic analogous of P(NDIOD‐T2) was the preparation of the monomer precursor, more precisely, an isolation of a desirable 2,6‐dibromo‐adduct from a complex mixture of isomeric products forming upon the bromination of naphthalenedianhydride^[^ ^57^ ^]^ (Figure S1, Supporting information). To overcome this obstacle, the bromination step was optimized to selectively obtain the desired isomer. This allowed avoiding tedious column chromatography separation steps and preparing the monomers in a total isolated yield of ≈60%. P(NDIOD‐T2) and ionic polymers were synthesized by a well‐established Stille polymerization.^[^ ^30^, ^58^ ^]^ The ammonium groups were introduced in a polymer‐analogous transformation by quaternizing amino groups. The identity of the synthesized polymers was confirmed by ^1^H‐NMR spectroscopy (Figures S2 and S3, Supporting information). Determination of molecular weight by using gel permeation chromatography (GPC) was possible only for the non‐ionic polymer P(NDIOD‐T2) whereas ionic polymers did not path through the chromatography column due to the strong interactions. For P(NDIOD‐T2), the GPC‐determined number and weight average molecular weights (Mn and Mw, respectively) were found to be Mn = 25 kg mol^−1^ and Mw = 42 kg mol^−1^. To evaluate molecular weights of the ionic polymers, dynamic light scattering (DLS) measurements were undertaken (Figures S4 and S5, Supporting information). Hydrodynamic radii, *R*_0_, of 8.8 ± 0.4 nm were found for P(NDIC3AI‐T2) and 17.2 ± 0.6 nm for P(NDIC3AI‐T2F). Assuming the polymers are approximated by a rigid rod model, these values correspond to molecular weights of 12.2 and 25 kg mol^−1^ for P(NDIC3AI‐T2) and P(NDIC3AI‐T2F), respectively. For P(NDIOD‐T2), *R*_0_ = 25 ± 3 nm was determined corresponding to the molecular weight of 38 kg mol^−1^, which corroborates well with the GPC data. The details of the calculation method from hydrodynamic radii to molecular weights are provided in Supporting Information.

### 2.2 Cyclic Voltammetry and Spectroelectrochemistry

Cyclic voltammetry (CV) combined with UV–vis spectroscopy was used for an in‐depth investigation of the redox behavior of the synthesized polymers. This in situ spectroelectrochemistry helps to correlate the redox state at the applied electrochemical potential to its characteristic absorption features.^[^ ^59^ ^]^ All measurements were performed in TBAPF_6_‐acetonitrile and TBAPF_6_‐dichloromethane electrolytes, which were carefully dried and deaerated beforehand. The potentials were given all versus the Fc/Fc^+^ redox couple.

**Figure 2:**
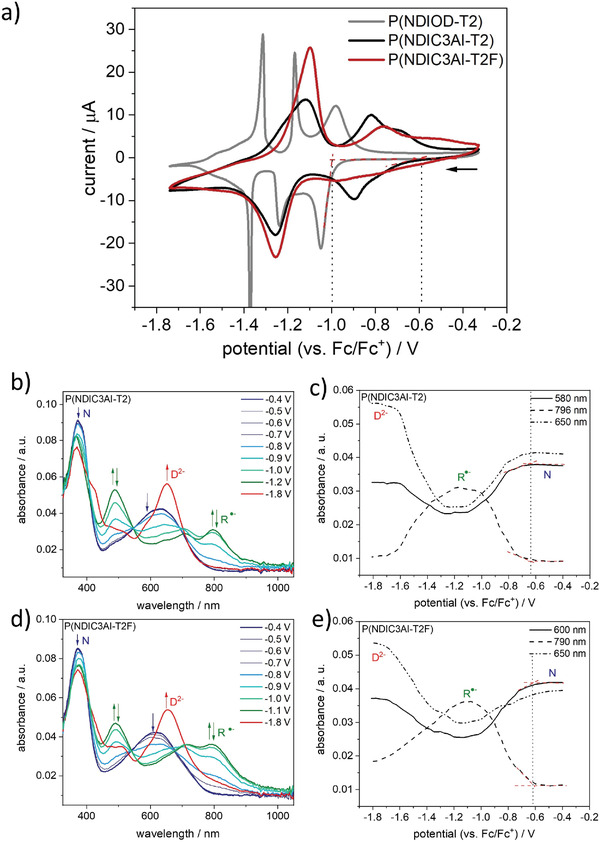
a) Cyclic voltammograms of P(NDIOD‐T2), P(NDIC3AI‐T2), and P(NDIC3AI‐T2F) films recorded on gold substrates in 0.1 M TBAPF_6_ in acetonitrile at a scan rate of 20 mV s^−1^ (the second cycles are shown). b–e) Spectra taken during the second cycle in the forward scans of b) P(NDIC3AI‐T2) and d) P(NDIC3AI‐T2F) in situ spectroelectrochemical experiments on ITO substrates in 0.1 M TBAPF6 in dichloromethane at a scan rate of 20 mV s^−1^. Intensities of three characteristic wavelengths are followed upon reduction and the trends are given in c) P(NDIC3AI‐T2) and e) P(NDIC3AI‐T2F). Reduction onsets are marked which are used for LUMO determination.

**Figure** **2**a compares the cyclic voltammograms of P(NDIOD‐T2), P(NDIC3AI‐T2), and P(NDIC3AI‐T2F) polymers. The CV of P(NDIOD‐T2) features two separated sets of sharp and well‐defined redox waves which are attributed to the first reduction from the neutral form N to a radical anion R^∙−^ state (≈ −1.0 to ≈ −1.3 V) and the second reduction from R^−^ to a dianionic form D^2−^ (≈ −1.3 to ≈ −1.7 V). The radical anion has characteristic absorption bands at 370, 490, and 800 nm and the dianion at 390 and 705 nm (Figure 2b). The peaks assignment can be done by in situ spectroelectrochemistry and is well‐documented in the literature.^[^ ^59^, ^60^ ^]^ It is well‐accepted that P(NDIOD‐T2) is a conjugated redox polymer with strong localization of the radical anion and dianion on the NDI units.^[^ ^59^, ^60^ ^]^ For P(NDIOD‐T2), the onset of the first reduction of −1.0 V versus Fc/Fc^+^ can be extracted from the CV and yields a LUMO of ≈ −4.1 eV, when assuming an offset of −5.1 eV with respect to the energy scale.^[^ ^59^, ^60^ ^]^

The CVs of P(NDIC3AI‐T2) and P(NDIC3AI‐T2F) are overall less defined and feature two partially superimposed broad redox waves for the first and the second reductions, which also suggests their redox polymer nature. An overall shift of the redox waves to more positive potentials is observed compared to P(NDIOD‐T2). The shape and position of the absorption peaks are quite similar for all polymers. In the case of P(NDIC3AI‐T2), the neutral species absorbs at 370 and 630 nm, the radical anion at 365, 490, and 795 nm and the dianion at 370 and 650 nm, respectively, see spectra at highlighted potentials in Figure 2b. For P(NDIC3AI‐T2F), the neutral species absorbs at 375 and 615 nm, the radical anion at 375, 490, and 800 nm and the dianion at 375 and 655 nm, respectively (Figure 2d).

The CV shifts can be quantified by determining the onset potentials of the first reduction. This can be done in the case of P(NDIC3AI‐T2) giving an onset of the first reduction of −0.6 V (vs Fc/Fc+). Since the first reduction is not clearly pronounced for P(NDIC3AI‐T2F), the determination of an onset from the CV cannot be easily done for this material. To overcome this issue, the spectral onsets were determined from the spectral evolution in Figure 2c–e. The obtained spectral onsets are positioned slightly below −0.6 V (vs Fc/Fc+) for both P(NDIC3AI‐T2) and P(NDIC3AI‐T2F) translating to a LUMO energy of around −4.5 eV. Both modified ionic polymers, therefore, exhibit onset potentials, which are shifted to more positive potentials compared to P(NDIOD‐T2). This means that both materials can be reduced easier and that they have an overall much higher n‐doping ability. One major difference between both new polymers is that they tend to have much stronger charge trapping behavior during multiple cycling. This can be seen from additional features in the CVs (see Figure S6, Supporting information) around 0 V suggesting that more positive potentials are needed for the re‐oxidation of the reduced polymer in the backward cycles. This can be very likely attributed to the chemical molecular structure of both P(NDIC3AI‐T2) and P(NDIC3AI‐T2F) polymers.

The incorporation of fluorine atoms into the main polymer chain and the introduction of the cationic ammonium side groups represent two powerful approaches to enhancing the n‐doping ability of the synthesized polymers. Both of these chemical strategies work in the same direction. Nonetheless, we propose different mechanisms to explain these phenomena. It is known that fluorine atoms are electron‐deficient substituents that influence the electron affinity (*EA*) of *π*‐conjugated systems.^[^ ^42^ ^]^ However, it is unlikely that the same mechanism is responsible for the effect of the tethered ammonium groups. The positively charged ammonium groups in P(NDIC3AI‐T2) and P(NDIC3AI‐T2F) are linked to the conjugated systems by a non‐conjugated propyl spacer, which diminishes their electron‐withdrawing effect. Instead, we believe that the high n‐doping ability of the cationic polymers is facilitated by the charge‐stabilizing interactions between the tethered immobilized ammonium groups and the negatively charged backbones in the n‐doped state. This was also proposed in the work of Tang et al.^[^ ^55^ ^]^

Doping always involves counterbalancing with ions. In a typical electrochemical reduction of the non‐ionic P(NDIOD‐T2), the radical anion and dianion species are charge‐compensated by TBA cations (accompanied by a solvent) coming from the supporting electrolyte. In the P(NDIC3AI‐T2) and P(NDIC3AI‐T2F) films, however, the tethered ammonium cations are likely to be involved in the charge compensating process as well. Although the excess of TBA cations from the electrolyte plays a role in counterbalancing the created charge in the CV experiments, the reduced states of P(NDIC3AI‐T2) and P(NDIC3AI‐T2F) may be stabilized with the tethered ammonium cations as well. The thus‐formed charge‐compensated doped states may benefit from an internal charge stabilization as manifested by their enhanced n‐doping ability.

### 2.3 Doping Studies

**Figure 3:**
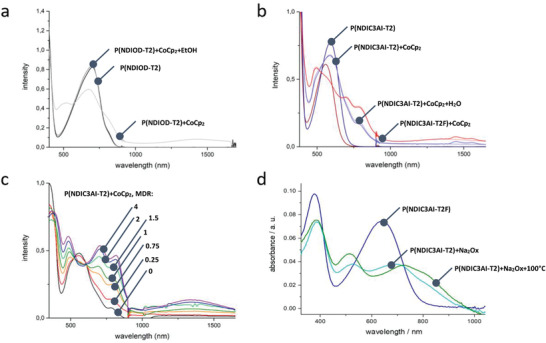
UV–vis absorption spectra of a) P(NDIOD‐T2) in DMSO in undoped (black) and CoCp_2_‐doped (light grey) state and after addition of ethanol (middle grey); b) P(NDIC3AI‐T2) in DMSO in undoped (dark blue), CoCp_2_‐doped at MDR = 1 (light blue), after addition of water (middle blue), the spectrum of P(NDIC3AI‐T2F) at MDR = 1 (red); c) P(NDIC3AI‐T2F) in DMSO with increasing CoCp_2_ equivalents: 0 (black), 0.25 (red), 0.75 (orange), 1.0 (green), 1.5 (light green), 2.0 (blue), 4.0 (purple); d) pristine (dark blue line) and Na_2_Ox‐doped P(NDIC3AI‐T2F) films (light blue) and the doped film after thermal annealing for 30 min at 100 °C.

Cobaltocene CoCp_2_, and sodium oxalate (Na_2_Ox) were used in this work as the n‐dopants.^[^ ^61^, ^62^, ^63^, ^64^ ^]^ According to the literature, the redox waves of CoCp_2_ are shifted by −1.3 V electronegatively relative to ferrocene (*E*_1/2_ = −3.8 eV).^[^ ^61^ ^]^ CoCp_2_ has an ionization energy (*IE*) of 4.0 eV.^[^ ^64^ ^]^ CoCp_2_ was chosen for its moderate doping strength and *IE* comparable with the *EA*s of the studied polymers. This allowed the detection of any differences in the n‐doping ability of the polymers. The exact oxidation potential of Na_2_Ox is more difficult to access as it strongly depends on the solvation (hydration) degree. Particularly, the CV of oxalates in solutions (e.g., in DMSO, +0.8 V vs SCE) does not explain the observed high n‐doping efficiency of Na_2_Ox.^[^ ^62^ ^]^ It was shown that oxalates act as n‐dopant only in a dry (solid) state, presumably in the form of nanoclusters.^[^ ^63^ ^]^ Furthermore, in contrast to CoCp_2_ which exhibits a broad scope as the n‐dopant, oxalates show their doping activity only against semiconductors having positively charged groups (i.e., CPCs). Moreover, oxalates are stable against oxidation in air, which makes them good candidates as n‐dopants in the open air as opposed to CoCp_2_ which should be handled in the glove box. **Figure** **3** compares optical absorption spectra of the P(NDIOD‐T2), P(NDIC3AI‐T2), and P(NDIC3AI‐T2F) solutions in the presence of CoCp_2_. Figure 3a shows absorption spectra of P(NDIOD‐T2) doped by CoCp_2_ at the molar doping ratio (MDR) of 4, recorded under air‐free conditions. Only at that high content of the dopant, a decrease of the absorption peaks from neutral P(NDIOD‐T2) is observed, along with an appearance of absorptions at 490 nm and 805 nm, assignable to the anion‐radical. In addition, a broad absorption peak extending between 1200 to 1600 nm is also observed. It is worth noting that such a broad peak was not detected in the spectroelectrochemistry investigations due to the wavelength limitation of our spectroelectrochemistry setup. However, this band should also be assigned to the anion‐radical rather than to the dianion because it appears together with other absorption peaks inherent to the radical anion (Figure 3c). At low contents of the dopant, such as at MDR = 1, the doping of P(NDIOD‐T2) with CoCp_2_ is barely observed. After opening the cuvette for 5 min, the signals of the n‐doped P(NDIOD‐T2) were degraded and the signals of the neutral polymer were restored indicating the environmental instability of the n‐doped P(NDIOD‐T2). The addition of a micro‐droplet of ethanol or water to the solution of the n‐doped P(NDIOD‐T2) also deteriorated the doping. In the film state, the cobaltocene‐doped P(NDIOD‐T2) spin‐coated at ambient conditions showed no signs of doping.

Figure 3b compares absorption spectra of CoCp_2_‐doped P(NDIC3AI‐T2) (blue line) and P(NDIC3AI‐T2F) (red line) at MDR = 1. The doping led to new absorptions peaking at 490, 705, and 815 nm as well as the infrared band at 1350 nm. A comparison of the absorption spectroscopy and spectroelectrochemistry data allows for identifying the formation of anion radicals under these conditions. The intensity of the anion‐radicals of P(NDIC3AI‐T2F) compared to P(NDIC3AI‐T2) is higher by a factor of 2.1, highlighting the higher n‐doping efficiency for the fluorinated polymer.

The environmental stability of the n‐doped P(NDIC3AI‐T2) and P(NDIC3AI‐T2F) was investigated. As evident from Figure 3b, the addition of a large excess of water to solutions of n‐doped P(NDIC3AI‐T2) has no influence on the spectra of the doped polymers (Figure 3b, a light‐blue line corresponds to the doped P(NDIC3AI‐T2) in the presence of water). This stability is striking taking into account that cobaltocene itself decomposes in water.

Figure 3c shows spectra of P(NDIC3AI‐T2F) solutions at different MDRs. These spectra obtained at the highest MDR = 4 resembles the spectrum obtained by electrochemical reduction at −1 V for the same polymer indicating a nearly complete conversion of P(NDIC3AI‐T2F) into the R^∙−^ form. Interestingly, the formation of the D^2−^ form of the polymers was not observed even at a large excess of the dopant. This could be explained by the more electronegative position of the D^2−^ peak (onsets of the second reduction peaks for both polymers lie at −4.15 eV) relative to the IP value of CoCp_2_ at 4 eV.

The data discussed so far are related to the doping by CoCp_2_ which required glove box conditions. We now describe the doping with Na_2_Ox, the attractive feature of which is that the doping could be achieved from water, due to its high solubility and stability in water. A few important peculiarities of Na_2_Ox have to be considered. First, the high doping strength of Na_2_Ox is exhibited only in the solid state, when nanoclusters having a high IP are formed, whereas in the hydrated state the IP is low. Consequently, the addition of water solution of Na_2_Ox to solutions of P(NDIC3AI‐T2) or P(NDIC3AI‐T2F) in DMSO causes no changes in absorption spectra reflecting the absence of the doping in solution state. Second, an application of a mixed solution doping method for the preparation of the doped films is challenging for polymers insoluble in water because of the low solubility of Na_2_Ox in solvents good for polymers. However instead, the use of the sequential deposition method is straightforward because most organic semiconductors are water insoluble. A drawback here is that the doping degree is difficult to control or vary. Finally, Na_2_Ox could be easily applied for the doping of polymers bearing cationic groups, thanks to ionic interactions, whereas involvement in the doping of other polymers, especially hydrophobic ones, is challenging.

To prepare the doped films, a droplet of a solution of Na_2_Ox in water was placed onto P(NDIC3AI‐T2) or P(NDIC3AI‐T2F) films for several seconds, and the films were dried by spin‐coating. By applying a similar spin‐coating procedure, the doped films were subsequently washed with water several times to remove excess of Na_2_Ox and any mobile ions.

Figure 3d shows representative absorption spectra of the undoped P(NDIC3AI‐T2F) film (dark blue), and the film after the doping and washing step (light blue) and doped film, which was thermally annealed for 15 min at 100 °C (green line). A high‐yield formation of the anion‐radical form of P(NDIC3AI‐T2F) upon doping is evident by the appearance of the characteristic absorption peaks. Importantly, the formation of the doped form does not require heating, as seen from the comparison of the blue and green lines, however, the thermal annealing slightly increases the doping degree.

It should be emphasized that some variations in the doping procedure, such as the application of different concentrations of the dopant, time for exposure of the dopant to the polymer film as well as variations in the water‐washing step had very little influence on the spectra of the doped films. The observed invariance of the doping results to the doping conditions indicates the fast ion exchange and strong binding of the oxalate ions to the cationic centers along the polymer chains. Studies in X‐ray photoelectron spectroscopy (XPS) were performed to determine the content of iodine anions before and after doping. These studies revealed that only ≈18% of the initial iodine amount remained after the doping, which is consistent with the formation of the self‐compensated doped states (Figures S8, and S9, Supporting information).

**Figure 4:**
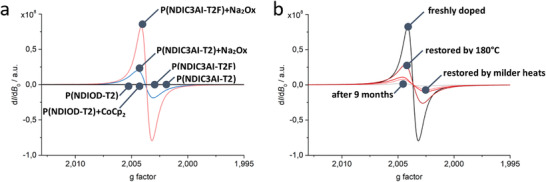
EPR spectra of a) Na_2_Ox‐doped films of P(NDIC3AI‐T2) (light blue) and P(NDIC3AI‐T2F) (pink). Undoped P(NDIC3AI‐T2), P(NDIC3AI‐T2F), and P(NDIOD‐T2) films, as well as doped P(NDIOD‐T2) films exhibit no EPR signals (black line). b) Na_2_Ox‐doped film of P(NDIC3AI‐T2F) (black), after storing for 9 months (grey), after subsequent heat treatment at 120 °C for 15 min (red) and 180 °C for 15 min (dark red); the line in between, the pink‐colored one, corresponds to less intensive heat treatment.

Electron paramagnetic resonance (EPR) studies allowed a quantitative evaluation of the doping efficiency. In accordance with the above‐discussed experiments, films of the reference polymer P(NDIOD‐T2) doped at ambient conditions by CoCp_2_, exhibited no EPR signals. In contrast, strong EPR signals were recorded for P(NDIC3AI‐T2) and P(NDIC3AI‐T2F) films doped with Na_2_Ox under ambient conditions. P(NDIC3AI‐T2F) exhibited a factor of ca. two times stronger EPR signal compared to P(NDIC3AI‐T2) (**Figure** **4**).

### 2.4 Conductivity

**Table** **1** collects the conductivities of P(NDIOD‐T2), P(NDIC3AI‐T2F), and P(NDIC3AI‐T2) doped films by different dopants.

**Table 1:** Conductivity results

Polymer	With CoCp_2_ [S cm^−1^]	With Na_2_Ox [S cm^−1^]	With sodium triflate [S cm^−1^]	With sodium citrate [S cm^−1^]
P(NDIOD‐T2)	< 1 × 10^−7^	‐	n/m^b)^	n/m^b)^
P(NDIC3AI‐T2)	9.29 × 10^−5^	1.30 × 10^−3^	n/m^b)^	n/m^b)^
P(NDIC3AI‐T2F)	4.69 × 10^−3^	2.51 × 10^−2^ (2 × 10^−1^)^a)^	2.2 × 10^−3^	3.1 × 10^−3^

^a)^ in vacuum

^b)^ n/m = not measured

For the CoCp_2_‐doped films, prepared and measured at ambient conditions, P(NDIC3AI‐T2F) exhibited a conductivity of 5 × 10^−3^ S cm^−1^, whereas the non‐fluorinated polymer P(NDIC3AI‐T2) showed an order of magnitude lower conductivity (10^−4^ S cm^−1^). The Na_2_Ox‐doping was generally more efficient and conductivities of 0.02 S cm^−1^ for P(NDIC3AI‐T2F) and 0.005 S cm^−1^ for P(NDIC3AI‐T2) were measured for Na_2_Ox‐doped films at ambient conditions. Interestingly, for P(NDIC3AI‐T2F) films prepared at ambient conditions but measured under a high vacuum, an order of magnitude higher conductivity of 0.2 S cm^−1^ was observed. This result can be explained by reversible trapping of a fraction of P(NDIC3AI‐T2F) polarons by environmental O_2_(H_2_O)_2_ species, as proposed by Brédas et al., and their recovery in vacuum.^[^ ^26^, ^27^ ^]^ Since the LUMO of P(NDIC3AI‐T2F) lies much below the reduction potential of O_2_(H_2_O)_2_ of −3.6 eV, the deactivation of polarons is a reversible process. It is in contrast to high‐lying LUMO OSCs, n‐polarons of which degrade irreversibly by a single‐electron transfer to O_2_(H_2_O)_2_.

The reference CoCp_2_‐doped P(NDIOD‐T2) prepared and measured at ambient conditions showed conductivity of 10^−7^ S cm^−1^, which is only slightly higher than the conductivity of the undoped P(NDIOD‐T2). To study the influence of the number of negative charges present in the dopant on their doping ability, the doping of P(NDIC3AI‐T2F) films with sodium triflate and sodium citrate was also studied. While oxalate is produced from dibasic acid, these salts are produced from monobasic trifluorosulfonic and tribasic citric acids. As seen in Table 1, these salts are efficient dopants, although they produced one order of magnitude lower conductivity. As such, it can be concluded that the “valence” of the dopants does not play a decisive role in doping efficiency.

**Figure 5:**
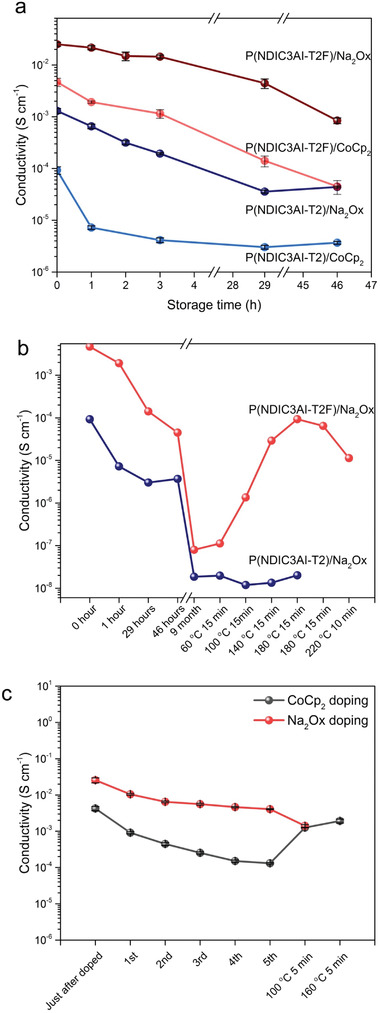
Changes in the conductivity of differently doped P(NDIC3AI‐T2F) and P(NDIC3AI‐T2) films: a) occurring upon the storage at ambient conditions, b) during long‐term storage followed by heat, c) conductivity of CoCp_2_ and Na_2_Ox‐doped P(NDIC3AI‐T2F) as a function of a number of water‐washing steps followed by the heat recovery.

The high water stability of the Na_2_Ox‐doped P(NDIC3AI‐T2F) and P(NDIC3AI‐T2) films was further highlighted in the water washing experiments. The water‐washing procedure was applied several times and conductivity measurements were performed after each washing step. As seen from **Figure** **5**c, each washing step caused a relatively modest decrease in conductivity and eventually saturated at an order of magnitude lower level after the fifth washing step. A visual inspection of the water‐treated films revealed significant corruption and a decrease in thickness suggesting that removal of the dopant and the polymer, rather than the dedoping is the main reason for the decrease in conductivity.

While the P(NDIC3AI‐T2F) and P(NDIC3AI‐T2) doped films showed lower conductivity at prolonged storage, they exhibited much higher stability than previously reported polymers having the same main chain but bearing water‐solubilizing side groups. In that work, the conductivity of the N‐DMBI‐doped p(gNDI‐gT2) dropped 3 orders of magnitude in 30 min of exposure to ambient conditions.^[^ ^64^ ^]^ We attribute the higher stability of our doped polymers to the stabilizing action of the cationic side chains.

To further investigate the environmental stability of the n‐doped polymers, conductivity measurements were conducted for samples stored in an open box covered with filter paper. As seen in Figure 5, the highest stability was exhibited by the Na_2_Ox‐doped P(NDIC3AI‐T2F) film. Its conductivity changed insignificantly within several days decreasing one order of magnitude after one‐month storage. A similar trend was observed for other combinations (CoCp_2_‐doped P(NDIC3AI‐T2F) and P(NDIC3AI‐T2) doped with Na_2_Ox or CoCp_2_), however, they exhibited systematically lower conductivity. A decrease of the conductivity in Na_2_Ox doped P(NDIC3AI‐T2F) films below 10^−6^ S cm^−1^ was observed in samples stored at ambient conditions for 9 months. The storage reduced the EPR signal to 6% of the initial signal intensity (Figure 4b). Interestingly, thermal annealing for 15 min at 180° of the aged P(NDIC3AI‐T2F) samples recovered their conductivity up to the 10^−3^ S cm^−1^ level. The annealing also recovered ≈85% of the initial EPR signal intensity (Figure 4b).

**Figure 6:**
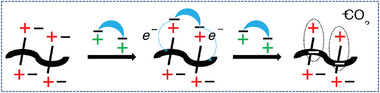
Schematic representation of the doping mechanism hypothesis for the n‐doping of ionic polymers by Na_2_Ox involving: i) the ion exchange step when two halogen anions are replaced by the oxalate dianion, accompanied by the release of two sodium halogenide molecules; ii) the two‐electron transfer process from the oxalate dianion to the polymer backbone, accompanied by a release of the carbon dioxide molecule.

It should be emphasized that only a high‐temperature treatment, but not the vacuum treatment at room temperature (*RT*) recovered the conductivity in the aged samples. This shows that the degradation mechanism involves something else than a reversible complexation of charge carriers with oxygen or water, as observed in freshly prepared samples. Instead, we propose that irreversible oxidation of the n‐doped states is responsible for the degradation in the long‐term aged samples. To understand the thermal recovery mechanism, the doping mechanism must be considered. We tentatively propose that the doping with Na_2_Ox is a two‐step process, which involves; i) an exchange of intrinsic halogen anions onto Ox^2–^ dianions, followed by ii) a full electron transfer (ET) from Ox^2–^ to the NDI‐based monomer unit of the cationic polymers (**Figure** **6**).

According to the hypothesis, each oxalate dianion is able to transfer two electrons producing a gaseous carbon dioxide by‐product, although the evolution of the carbon dioxide was not proved experimentally. Another by‐product forming during the ion‐exchange step is a water‐soluble sodium halide, which should be removed from the doped polymer film during the water washing step. Consequently, we attribute the deterioration of the conductivity upon the long‐term storage to irreversible oxidation of the doping charges.

To explain the conductivity recovery, we propose that not all Ox^2−^ attached to the cationic polymer undergo the ET during the sequential doping of the film (primary doping process) and a fraction of unreacted Ox^2−^ is stored in the doped film as “latent” dopants. It is noteworthy that the primary doping proceeds efficiently at *RT*. This suggests that the “latent” dopants may also be activated at *RT*, for example, to “heal” doping states, which were deactivated during the operation or storage by environmental oxygen. As the secondary doping process proceeds on its own (i.e., does not require external actions), it may contribute to enhanced environmental stability of our Na_2_Ox‐doped cationic polymers. Why the recovery of the long‐term stored samples needs heat treatment is not fully clear to us. A possible interpretation might be that heat is to enhance diffusion in the film, for example, for delivering the latent dopants stored in amorphous parts into more crystalline areas, which define conductivity.

Regarding the accumulated dopant amount in the doped polymer films, it is worth noting that i) XPS measurements showed an 82% degree of the I^−^ to Ox^2−^ exchange upon doping (Figure S2, Supporting information), ii) each monomer unit bears two cationic groups and therefore is able to capture one oxalate molecule, iii) this amount of the dopant should be sufficient for the formation of the dianion form of the doped polymer with the 82% yield, however, iv) only the radical form of the doped polymers is formed. As such, simple estimations show that almost half of the accumulated dopant molecules are not consumed upon the primary doping and may act as latent dopants.

### 2.5 Temperature‐Dependent Conductivity

**Figure 7:**
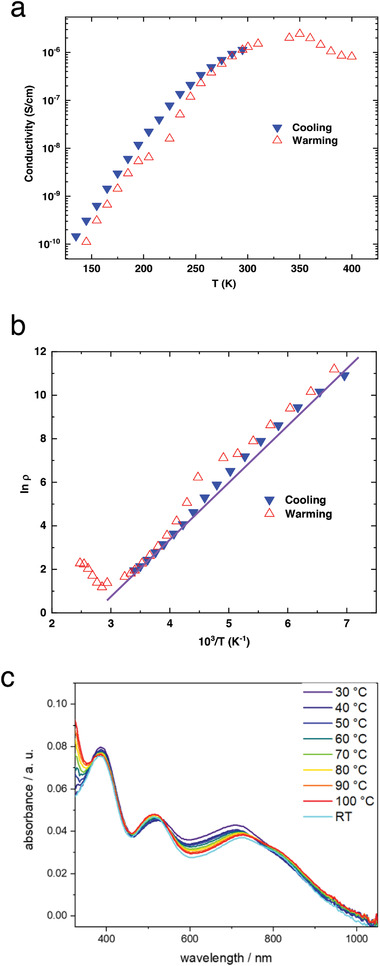
a) Temperature‐dependent conductivity of thin (80 nm) P(NDIC3AI‐T2F) film doped by Na_2_Ox observed in the 150–400K temperature range; b) *lnρ* versus 1/*T* dependence and its linear fit which were used to estimate the activation energy *E_a_*; c) temperature dependence of the absorption spectra of the Na_2_Ox‐doped P(NDIC3AI‐T2F) film.

To study the charge transport mechanism, temperature (*T*) dependent conductivity (*ρ*) measurements were undertaken. A strong decrease of the conductivity with the cooling from 300 to 150 K, was observed (**Figure** **7**a), which is characteristic of a thermally activated hopping mechanism. Similar behavior was observed with the heating from 150 to 300 K implying the reversibility of this behavior. Further heating of the sample from 300 to 350 K, revealed that the conductivity increases following similar behavior as below 300 K. However above 350 K, we observed a rapid decrease of the conductivity. Since the conductivity of the sample was not recovered after cooling down to 300 K, this decrease was most probably caused by the irreversible degradation of the sample at high temperatures.

The activation energy *E_a_* was estimated from the linear fit of the *lnρ* versus 1/*T* dependence (Figure 7b). We observed *E_a_* of 240 meV in the temperature range of 150–350 K which is much larger compared to other OSCs with high conductivity. For comparison, archetypical poly(3‐hexylthiophene‐2,5‐diyl) (P3HT), which has a comparable conductivity in the doped state, has the *E_a_* of 54–69 meV.^[^ ^65^ ^]^ High *E_a_* of 200 meV was previously measured for P(NDIOD‐T2) doped with pentamethylrhodocene dimer under inert conditions, however, that system exhibited 2–3 orders of magnitude lower maximum conductivity level.^[^ ^66^ ^]^ Generally, high activation energy in the NDI‐based polymers could be attributed to an inherent tendency of NDI repeat units to strongly localize polarons, which causes a high contribution to the hopping of the charge transport mechanism^[^ ^66^ ^]^ at expense of the charge transport through delocalized polarons.^[^ ^67^ ^]^ We suggest that the presence of cationic side chains in our polymers is an additional factor localizing electrons on the NDI repeat units due to Coulomb attractions, which leads to an extraordinarily high *E_a_*.

### 2.6 Field Effect Transistor Measurements

Attempts to realize a bottom contact‐bottom gate FET was done for undoped P(NDIC3AI‐T2F) and Na_2_Ox‐doped P(NDIC3AI‐T2F). In devices with undoped P(NDIC3AI‐T2F), a negligibly low current was observed in the gate voltage varied from −100 to 100 V, while for Na_2_Ox‐doped P(NDIC3AI‐T2F), a high current was observed with no possibility to switch the device off or even decrease the current. The possible reason for the absence of the field effect will be discussed below.

### 2.7 UV–vis Spectroscopy

To verify whether the observed temperature dependence is indeed due to the temperature‐activated electron‐hopping or is a result of increased doping efficiency at elevated temperature, temperature‐dependent UV‐near‐IR absorption spectroscopy measurements of the doped films were undertaken. UV‐near‐IR absorption spectroscopy provides useful information about the concentration of charge carriers. We assumed, that if the tenfold increase of the conductivity observed upon heating from 300 to 350 K is due to an increased capability of oxalate to dope P(NDIC3AI‐T2F), a corresponding heat‐induced increase in the intensity of absorptions inherent to polarons must be observed. As seen in Figure 7, only a slight red shift of the absorption inherent to R^∙−^ of P(NDIC3AI‐T2F) along with a < 5% increase of the absorption intensity at 850 nm is observed with the temperature rise from 300 to 400 K. Such changes cannot explain the observed 1000% temperature‐induced increase of the conductivity and likely, these changes are given by a thermal reorganization of the film.

### 2.8 GIWAXS

**Figure 8:**
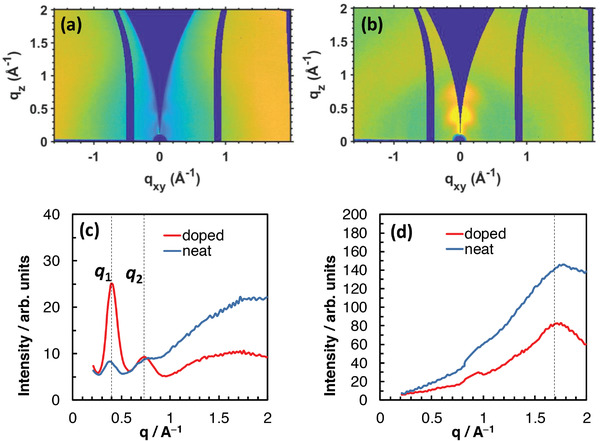
GIWAXS data of a) neat P(NDIC3AI‐T2F) film and b) Na_2_Ox‐doped P(NDIC3AI‐T2F) film. Line‐cut profiles of the two GIWAXS data over c) out‐of‐plane (azimuthal range *χ* of −20° to 20°) sector and d) in‐plane (azimuthal range *χ* of 80° to 90°) sector. Characteristic Bragg peaks are indicated by dashed lines.

To further understand the origin of the high conductivity of P(NDIC3AI‐T2F) doped by Na_2_Ox, the molecular packing, crystallinity, and chain orientation of neat and doped films were investigated using grazing‐incidence wide‐angle X‐ray scattering (GIWAXS). As can be seen in **Figure** **8**a, the neat P(NDIC3AI‐T2F) polymer film showed weak diffraction indicating its poor order. The doping was performed by coating the polymer films with Na_2_Ox solution. As revealed by XPS, this procedure led to an 82% exchange of intrinsic iodine anions by oxalate anions which indicates a high doping degree. This has induced a much more ordered structure of the doped film as evidenced by the stronger discernible diffraction peaks (Figure 8b). To analyze the GIWAXS data, integrations of the intensity over out‐of‐plane (azimuthal range *χ* of −20° to 20°) and in‐plane and (azimuthal range *χ* of 80° to 90°) sector cuts were performed, as shown in Figure 8c,d, respectively. For the neat film, the out‐of‐plane line‐cut profile exhibited a weak Bragg peak at *q*_1_ = 0.38 Å^–1^, which corresponds to a spacing of *d* = 16.6 Å, and a diffraction feature at *q* ≈0.73 Å^–1^. The observed broad reflection that extends between *q* = 1.00 and 2.00 Å^–1^ indicates a predominantly amorphous packing of the P(NDIC3AI‐T2F) chains. The in‐plane line‐cut profile showed a weak reflection at *q* = 1.80 Å^–1^ due to the *π*–*π* stacking with a spacing of 3.48 Å. For the doped film, the out‐of‐plane line‐cut profile exhibited two pronounced Bragg peaks at *q*_1_ = 0.40 Å^–1^ and at *q*_2_ = 0.73 Å^–1^. Notably, the spacing corresponding to the first Bragg peak has decreased to 15.7 Å upon doping. However, the *π*–*π* stacking spacing has increased to 3.65 Å after doping as revealed by the in‐plane line‐cut profile of the doped film. To evaluate the change in crystallinity of the P(NDIC3AI‐T2F) polymer upon doping, we compare the area under the *q*_1_ peak of the doped film relative to that of the neat film. A ratio of 7.5 is found confirming the positive impact of the Na_2_Ox molecules on the crystallinity of the P(NDIC3AI‐T2F) polymer.

In general, morphological changes observed upon the incorporation of dopants into *π*‐conjugated polymers are highly dependent on the nature of the polymer/dopant pair and the amount of the dopant. Most frequently, only a slight increase of the order with almost no change in crystallinity is observed at low content of the dopant (< 10%), whereas high concentrations of dopants lead to a strong increase in the disorder.^[^ ^39^, ^42^, ^68^, ^69^, ^70^, ^71^ ^]^ This is a rather natural effect of weakly interacting “foreign” molecules dispersed in semicrystalline polymers. A drastic increase in the crystallinity of the polymer in the presence of large amounts of dopant, observed herein, is a rather unusual phenomenon. It points to specific intermolecular interactions and the formation of ordered complexes between the NDI groups and the Na_2_Ox dopant molecules.

### 2.9 Theoretical Calculations

To better understand experimental results, theoretical calculations were undertaken. We modeled monomeric units of respective polymers instead of the whole chains, to save calculation time. We suggest that this approximation still provides reliable trends because the most important effects studied in this work are expected to come from side groups and should not be too strongly affected by a decreased conjugation length. In addition, a peculiarity of the NDI‐based polymers is that their properties are less sensitive to variation of conjugation length due to the efficient localization of electrons by the NDI group. To model the charged structures, propyl side groups having quaternized trimethyl ammonium head and variable counterions were introduced into nitrogen atoms of imide groups. As the reference named NDIC3‐T2, a repeat unit of P(NDIOD‐T2) was modeled, in which ‐C_20_H_41_ groups were replaced by shorter –C_3_H_7_, to save calculation time.

As shown in the literature, ionic polythiophenes^[^ ^72^, ^73^, ^74^ ^]^ and poly(fluorene‐phenylene)s^[^ ^75^, ^76^ ^]^ are theoretically the most studied *π*‐conjugated macromolecules with ionic side groups. Their conformational properties, aggregation, as well as specificity of the counterion or surfactant bindings, studied by molecular dynamics simulations were the main focus of these publications.^[^ ^72^, ^73^, ^74^ ^]^ In addition, optical,^[^ ^75^ ^]^ or electronic^[^ ^76^ ^]^ properties were characterized within the density functional theory approach. On the other hand, works on the calculation of adiabatic ionization energies and electron affinities are scarce.^[^ ^76^ ^]^ We believe that the present study is the first work that shows the calculated adiabatic electron affinities for NDI‐based ionic polymers to shed light on the effects of their n‐doping.

*IE* and *EA* are important parameters for understanding the charge transfer, conductivity doping, and charge transport phenomena in organic semiconductors. Experimentally, *IE* and *EA* can be determined via UV photoelectron spectroscopy and inverse photoemission spectroscopy, respectively, or by electrochemistry. For an accurate theoretical prediction of *IE*s, *EA*s, and transport gaps from first principles, the “state‐of‐the‐art” method is s many‐body perturbation theory within the GW approximation (GWA).^[^ ^77^, ^78^, ^79^ ^]^ However, GWA is computationally much more demanding than density‐functional theory (DFT) so the computing of large molecules, which is of interest in this study, is a great challenge. In general, the *EA* of a molecule is the amount of energy released when an electron is attached to a neutral molecule to form a negative ion. Consequently, the *EA* is a difference of full energies between the anion (the molecule with the added electron) and the neutral molecule. A simplified way to compute *IE* and *EA* is based on the Koopmans’ theorem,^[^ ^80^ ^]^ which states that *IE* and *EA* of a molecular system are equal to the negative of the orbital energy of the highest occupied molecular orbital (HOMO) and lowest unoccupied molecular orbital (LUMO), respectively. A drawback of this approach is that it does not consider relaxations (e.g., geometrical, electronic), which take place after the charging of molecules. Nevertheless, it provides, in many cases, reliable data and allows the determination of trends in a series of molecules having systematically varied structures.^[^ ^79^ ^]^

The most important calculated values are given in **Table** **2**. We start our analysis by examining the LUMO energies of structures in the neutral (i.e., undoped) state (Column 3). For the non‐ionic reference NDIC3T2, which has two propyl side groups, LUMO of −3.82 eV was obtained (Table 2, entry #1). A compound having two non‐quaternized amino side groups, shown in entry #2, has almost the same LUMO energy as the alkyl‐substituted reference NDIC3‐T2 (−3.83 eV). Substitution of two hydrogens in the bithiophene bridge by two fluorine atoms decreases the LUMO by ≈0.16 eV, reflecting an energy‐stabilizing effect of the electron‐withdrawing groups. Entries #3–6 give the LUMO energies for the structures with quaternized amino side groups and having different counterions (Cl^−^ and I^−^), with and without fluorine substituents in the bithiophene bridge. The calculations predict a modest stabilization of LUMOs in molecules with quaternized ammonium groups and different counterions (0.29 and 0.09 eV, for Cl^−^ and I^−^, respectively, Table 2, column 3, #1 vs #3 and #5). A comparison of the calculation results for the structures with quaternized and non‐quaternized side groups suggests that the LUMO energy is influenced through an electrostatic stabilization mechanism rather than a self‐doping^[^ ^81^, ^82^ ^]^ mechanism. We assumed that in the undoped molecule, the positive charge on the ammonium group is largely compensated by the oppositely charged counter anion, which may account for a reduced charge‐stabilizing influence of the ammonium group. We further suggested that the calculations based on Koopmans’ theorem, which for the evaluation of *EA* deal with undoped structures, may give especially inaccurate results in the case of structures having ionizable groups. It is because such structures, receiving the negative charge upon the doping, may undergo significant relaxations driven by electrostatic interactions.

**Table 2:** The LUMO energies and the adiabatic electron affinities of the calculated samples

Col1^a)^	Col2: Name of the structure	Col3: LUMO [eV]	Col4: *EA* [eV]^a)^
#1	NDIC3‐T2	−3.82	2.36
#2	NDIC3A‐T2 (not quaternized)	−3.83	2.38
#3	NDIC3A‐T2(2Cl^−^)	−4.11	2.88
#4	NDIC3A‐T2F(2Cl^−^)	−4.27	3.13
#5	NDIC3A‐T2(2I^−^)	−3.91	2.79
#6	NDIC3A‐T2F(2I^−^)	−4.05	2.93
#7	NDIC3A‐T2(Cl^−^)	−5.71	4.52
#8	NDIC3T2(Na^+^)	−6.49	5.26

^a)^ adiabatic *EA*s are given by the difference between the energy of the neutral molecule at its most stable geometry, and of the anion, also at its most stable conformation. These values are shown in column 4 (Col4).

To verify this hypothesis, adiabatic electron affinities (Column 4, Table 2) were computed as a difference between the energy of a corresponding neutral molecule, and of the anion formed upon the addition of an electron to the molecule, both taken in their most stable conformations. For the non‐ionizable reference NDIC3‐T2 and its fluorine‐containing analogous NDIC3‐T2F, EAs of 2.36 and 2.58 eV respectively were obtained. Ionizable structures show the same trend, namely that the incorporation of fluorine atoms increases *EA*s by ≈0.15 eV (e.g., entry 3 vs 4). These results demonstrate that the calculation approach reliably reproduces the effect of the electron‐withdrawing groups (F) on the *EA*. Incorporation of non‐quaternized alkyl amino side groups does not change *EA* (Table 2, column 4, #2 vs #1), which reproduces calculation results based on the Koopmans’ theorem (Table 2, column 3, #2 vs #1).

In contrast, for structures with quaternized ammonium side groups, the calculations predict a significant increase of the adiabatic *EA*s by 0.52 and 0.41 eV for the structures with Cl^−^ and I^−^ counterions, respectively (Table 2, column 4, #1 vs #3 and #5), which is larger than the LUMO increase predicted in the calculations based on the Koopmans’ theorem. More importantly, the calculations of adiabatic *EA*s reproduce well the experimentally observed significantly higher n‐doping ability of polymers with ionizable side groups (P(NDIC3AI‐T2) and P(NDIOD‐T2F) compared to the non‐ionic polymer P(NDIOD‐T2). These results show that the use of computationally more demanding approaches based on the calculation of adiabatic *EA* is strictly needed for the structures having ionizable groups, whereas the structures having no ionizable groups could be satisfactorily computed with less demanding calculations based on the Koopmans’ theorem.^[^ ^80^ ^]^

To understand the doping‐induced stabilization effects occurring in the *π*‐conjugated molecules bearing cationic side groups, the distribution of charges was evaluated for the undoped and doped states and particular attention was paid to the charges on counter anions. For the neutral (undoped) molecule #3, the charge of the counter anion Cl^–^ is −0.61 *e* (where *e* is the elementary charge), reflecting a not fully ionic type of bonding between the ammonium group and the anion through a partial donation/redistribution of the electron density from Cl^−^ to NR_4_^+^. The n‐doping substantially increases the negative charge on the Cl^−^ to −0.74 *e*. We attribute the observed increase of the negative charge on the counter anion to a sequential shift of electron density from NDI, which received an additional electron upon the doping, to the ammonium center and then, to Cl^−^. Thus, the ammonium group, on one side, stabilizes the n‐doped NDI core by withdrawing a part of the negative charge, and on the other side, reduces interactions with the counter anion. The observed doping‐induced increase of the charge on the counter anion reflects an increase in the ionic character of the bond between the counter anion and ammonium group, which is equivalent to a partial release of the counter anion.

**Figure 9:**
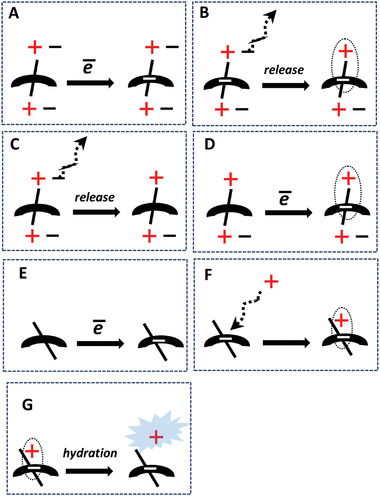
A scheme summarizing processes possibly occurring upon the n‐doping of ionic (reactions A–D) and neutral (reactions E–G) polymers. More details are given in the main text.

Reaction A (**Figure** **9**) represents doping of the structure having both intrinsic counter anions (also shown in entries #3–6, Table 2) with one counter anion undergoing release after the n‐doping (reaction B). However, the release of the intrinsic counterion prior to the n‐doping (e.g., NDIC3A‐T2(Cl^−^), entry #7, Table 2) is also feasible in polar solvents (process C).^[^ ^81^, ^82^ ^]^ As shown in our calculations, molecules, which receive a positive charge upon the counter anion release, increase their *EA*. After the n‐doping, NDIC3A‐T2(Cl^–^) transforms into the neutral structure, which corresponds to a highly stabilized charge‐compensated doped state, according to Tang et al.^[^ ^55^ ^]^ Thus, charge‐compensated doped states (products of reactions B and D, Figure 9) may be formed via two pathways, which differ in the sequence of the doping and counter anion release steps. The calculated *EA* of NDIC3A‐T2(Cl^−^) is 4.52 eV, which is 2.2 eV higher than the *EA* of the neutral molecule (entry#1, Table 2).

Figure 9E–G illustrates the n‐doping of non‐ionic reference P(NDIOD‐T2). It is a two‐step process, which involves charging negatively the polymer backbone (E) and compensation of the negative charge by a counter ion (F). To evaluate the influence of a positive charge on the *EA* of the non‐ionic molecule, structure #8 with sodium cation located nearby oxygen of the imide group of NDIC3‐T2 was also optimized. We found that NDIC3‐T2(Na^+^) has the *EA* value, which is even higher than the affinity of NDIC3A‐T2(Cl^−^) (5.26 eV, entry #8). At first sight, this result contradicts the experiment, which shows a much weaker n‐doping ability and lower stability of the n‐doped state for the non‐ionic polymer P(NDIOD‐T2) compared to the cationic polymers. For a correct interpretation of different stabilities of the n‐doped P(NDIC3AI‐T2) versus P(NDIOD‐T2) with regards to calculated EAs for NDIC3A‐T2(Cl^−^) versus NDIC3T2(Na^+^), the entropy factor must be considered. Particularly, it should be noticed that NR_4_^+^ in NDIC3AT2(Cl^−^) is covalently attached to the rest of the molecule and cannot be released under any circumstances, whereas Na^+^ cation in NDIC3T2(Na^+^), is bound by means of electrostatic interactions and can easily be released, especially in polar solvents (Figure 9G). This fact may explain an immediate degradation of the n‐doping of P(NDIOD‐T2) in the presence of traces of water. We postulate that highly polar water molecules tend to segregate in close vicinity to the most polar part of the doped P(NDIOD‐T2), that is, around Na^+^ cations and solvate them (which is equivalent to their release). Hydrated Na^+^ cations render a weaker stabilization effect on highly energetic anion radicals. While the counter cation release is an unfavorable process from the enthalpy standpoint, the energy loss can be compensated by the large hydration energy and due to a significantly increased entropy of the system.

**Figure 10:**
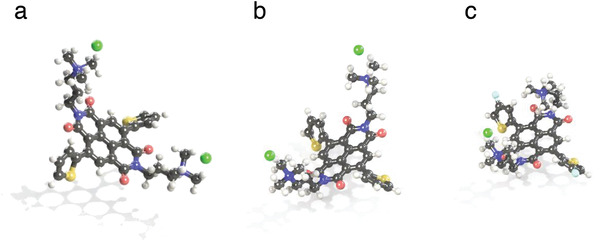
Optimized geometries of a) undoped NDIC3A‐T2(2Cl^–^), b) the n‐doped NDIC3A‐T2(2Cl^−^) having two counter anions, and c) the n‐doped NDIC3A‐T2F(Cl^−^) with one counter anion; as such, the structure (c) represents the Tang's charge‐compensated doped state.

To better understand the results of the calculations predicting stabilization of the negative doping charge by positive charges on the ionized side groups, the chemical structures of the calculated molecules were visualized. **Figure** **10** compares a) the undoped structure NDIC3A‐T2(2Cl^–^) and corresponding n‐doped structures having either b) two or c) one Cl‐ counter‐ions. While the a) undoped structure has symmetrically positioned side groups relative to the NDI‐T2 group in the middle representing the polymer backbone unit, the introduction of the doping charge on the backbone leads to molecular rearrangement, predominantly of the side group in the way that the quaternized ammonium center approaches significantly b) the NDI‐T2 group. This rearrangement illustrates the effect of electrostatic stabilization. Interestingly, the removal of one of the counter ions, which models the counterion release and the formation of the Tang's charge‐stabilized doped state,^[^ ^55^ ^]^ leads to further collapse of side groups toward the backbone in the way that now both side groups are involved in the stabilization. Indeed, the separation between the most distant carbons of the side chains for the NDIC3A‐T2 in its extended conformation prior to any geometry optimization is 19.4 Å. For the same molecule having optimized geometry, the distances are 16.1 and 15.8 Å for a) undoped and b) doped state, respectively, and 8.0 Å for the c) n‐doped NDIC3A‐T2F(Cl^–^) with one counter anion.

## 3 Conclusion

In this work, the n‐doping ability of three NDI‐based polymers, non‐ionic P(NDIOD‐T2) and polymers with cationic side groups P(NDIC3AI‐T2) and P(NDIC3AI‐T2F), was investigated. According to CV and spectroelectrochemistry data, the incorporation of cationic side groups strongly facilitates the reduction ability of the polymers (P(NDIC3AI‐T2) and P(NDIC3AI‐T2F) compared to P(NDIOD‐T2)). Both (P(NDIC3AI‐T2) and especially, P(NDIC3AI‐T2F), exhibits very broadened redox peaks, which extend strongly into the electropositive potential direction. The LUMO for the three polymers was evaluated at the level of −4.1 V for P(NDIOD‐T2) and below −4.4 V and −4.5 eV for P(NDIC3AI‐T2) for P(NDIC3AI‐T2F), respectively. Although the fractions of states with the highest electron affinity constitute minority fractions in both cationic polymers, they are very important in defining the electronic properties and conductivity of the whole polymer batches.^[^ ^83^ ^]^

Investigations of the n‐doping ability and conductivity of the polymers confirm this assumption. The highest n‐doping ability exhibits P(NDIC3AI‐T2F), which in the presence of a moderate‐strength n‐dopant cobaltocene could be near quantitatively doped into the radical‐anion state. While the n‐doped P(NDIOD‐T2) immediately degrades in air, the cationic polymers exhibit high water stability of their n‐doping states, but slowly oxidize with oxygen. The thermodynamic stability in water is consistent with the electrochemistry data, which show that a substantial fraction of states in P(NDIC3AI‐T2F) extends beyond the water reduction potential of −4.44 eV, which reflects the thermodynamic stability of the n‐doped P(NDIC3AI‐T2F) in water. The doping of P(NDIC3AI‐T2F) could be accomplished by using sodium oxalate solution deposited from water, which further highlights the exceptional stability of the n‐doped state. This is in contrast to previously reported n‐doped polymers, which exhibit only kinetic stability against water either due to a self‐encapsulation effect (e.g., tight packing of side groups slows down water penetration), or because the reaction with water occurs slowly in the absence of specific catalysts for water reduction.

The n‐doped P(NDIC3AI‐T2F) shows a high conductivity of up to 10^−2^ S cm^−1^ in an open environment and of 10^−1^ S cm^−1^ in vacuum. The decreased conductivity in air and restoration of the high conductivity in a vacuum is likely due to a reversible formation of complexes between negatively charged polarons and oxygen, which recover free polarons in vacuum due to the elimination of oxygen. In contrast, the n‐doped state of the non‐ionic P(NDIOD‐T2) could not be restored in this way. While short‐term reactions of polarons with oxygen are reversible, long‐term storage (months scale) of the n‐doped P(NDIC3AI‐T2F) causes more severe degradation so that conductivity could not be recovered in a vacuum anymore. However, conductivity in the long‐time aged Na_2_Ox‐doped P(NDIC3AI‐T2F) samples could partially be restored by heat and thermally‐annealed samples show a relatively high conductivity of 10^−3^ S cm^−1^. We believe that the recovery, in this case, is due to the presence in the sample of the “latent dopant” – oxalate anions – accumulated by cationic side groups, which could be reactivated by heat.

GIWAXS investigations revealed a drastic (7.5 times) increase in the crystallinity degree of P(NDIC3AI‐T2F) films upon the doping with Na_2_Ox, accompanied by a decrease of the out‐of‐plane distance from 16.6 to 15.7 Å and an increase of the *π*–*π* stacking spacing from 3.49 to 3.65 Å. While an exact molecular structure of the doped polymer is unknown, the observed contraction of the inter‐chain distance may indicate that bidentate oxalate anions interact strongly with the cationic groups stitching and compacting the polymer structure. The increase of the crystallinity usually has a favored effect on conductivity (providing that crystalline domains are well‐interconnected), although an increase of the *π*‐staking distance disfavors the charge transport.

Still another interesting property of Na_2_Ox‐doped P(NDIC3AI‐T2F) films is their exceptionally strong temperature dependence on electrical conductivity. Particularly, 5 orders of magnitude conductivity increase were observed in the doped P(NDIC3AI‐T2F) films upon the temperature rise of 250 K, which corresponds to a high activation energy of ≈300 meV.

In general, the positive dependence of the conductivity on the temperature is a signature of the charge‐hopping mechanism of the charge transport. To enable the charge hopping, activation energy is needed, which for many organic semiconductors lies below 100 meV. The unprecedentedly high activation energy in P(NDIC3AI‐T2F) is a combination of two factors – a strong localization of polarons on NDI and the action of cationic side chains, which strongly interact with the oppositely charged carriers to slow down their movement. Hence, additional activation energy is needed to overcome strong Coulomb attractions between moving electrons and cationic groups. This is in contrast with other organic semiconductors, for which the activation energy is needed predominantly for electronic reorganizations. Our result may be described in terms of strongly reduced electron mobility, which is decreased not due to a high disorder in the film (GIWAXS data reveal relatively high crystallinity of the doped P(NDIC3AI‐T2F)) but due to the presence of the cationic side chains acting as regularly placed charge traps for hopping electrons.

Thus, the cationic side groups cause a dual effect on the charge transport efficiency in the *π*‐conjugated polymer. From one side, they facilitate the n‐doping ability, by stabilizing the doped state and, in such a way, increasing the charge carrier concentration. On the other side, they act as charge traps, decreasing the effective electron mobility, which however could be overcome with heat to provide reasonably high conductivity. In other words, the retarding action of the cationic side groups on electron transportation is the price that one has to pay for achieving the facilitated n‐doping ability of the polymers, as well as for the water stability of the n‐doped state.

Theoretical calculations shed more light on the n‐doping ability. An absence of the LUMO‐stabilizing effect of the cationic groups was found in the undoped polymers. At the same time, the calculations correctly describe the LUMO‐stabilizing effect of electron‐withdrawing atoms incorporated directly into the *π*‐conjugated core, confirming the general validity of the calculation method. As such, a commonly used approach to estimate the electron affinity of a molecule as the LUMO energy taken with a reverse sign leads to especially inaccurate results for molecules with ionizable groups. We suggested that the stabilizing effect of the cationic groups can be satisfactorily reflected when relaxations of the ionizable molecule occurring after the n‐doping are considered. To this end, adiabatic electron affinities were computed as the difference between energies of a neutral molecule and of the corresponding anion formed upon the addition of an electron. This calculation approach reproduces well the experimentally observed significantly higher electron affinities of the ionizable compounds compared to the non‐ionic compounds (e.g., 2.88 eV for NDIC3A‐T2(2Cl^–^) vs 2.36 eV for NDIC3T2). We attributed the increased electron affinity effect, to an increased charge‐stabilizing influence of the ionizable ammonium group, which takes place, possibly, due to a “partial release” of the “intrinsic” counter anions. This assumption was confirmed by calculations, which reveal a substantial doping‐induced increase of the effective charge (from −0.61 to −0.74 *e*) of the chlorine counter anion. The molecule was also modeled in which one intrinsic counter anion is fully released, imparting the positive charge to the structure. After the n‐doping, it transforms into the neutral species, which corresponds to a highly stabilized charge‐compensated doped state. The calculated *EA* for the structure NDIC5A‐T2(Cl^–^) is 4.51 eV, which is 2.2 eV higher than the *EA* for the neutral molecule (entry #1). Geometry analysis of the model ionic molecules shows a significant doping‐induced “collapse” of ionic side groups, which manifests itself by a close approach of the positively charged ammonium center to a fragment of the conjugated system bearing the negative charge. This result reproduces a doping‐induced decrease of the lamellar spacing experimentally observed by GIWAXS measurements.

In conclusion, we have developed the n‐type conducting material P(NDIC3AI‐T2F) with enhanced stability of the n‐doped states for prospective “in‐water” applications. The present work brings important information for the understanding of the role of the ionic side group in the enhancement of the n‐doping ability of organic semiconductors. The obtained knowledge allowed designing n‐type organic semiconductors, which exhibit thermodynamic stability in water.

## Conflict of Interest

The authors declare no conflict of interest.

## Supporting information

Supporting Information

## Data Availability

The data that support the findings of this study are available from the corresponding author upon reasonable request.
