# Supplementary material for: Charge‐Compensated N‐Doped π‐Conjugated Polymers: Toward both Thermodynamic Stability of N‐Doped States in Water and High Electron Conductivity
Source: Adv Sci (Weinh). 2022 Sep 5;9(31):2203530. doi: 10.1002/advs.202203530 (PMC9631074; doi:10.1002/advs.202203530)
Supplement: Supplementary file 1 — Supporting Information [file ADVS-9-2203530-s001.pdf]

ADVANCED SCIENCE
Open Access

Supporting Information

for *Adv. Sci.*, DOI 10.1002/advs.202203530

Charge-Compensated N-Doped π-Conjugated Polymers: Toward both Thermodynamic Stability of N-Doped States in Water and High Electron Conductivity

Fabian Borrmann, Takuya Tsuda, Olga Guskova, Nataliya Kiriy, Cedric Hoffmann, David Neusser, Sabine Ludwigs, Uwe Lappan, Frank Simon, Martin Geisler, Bipasha Debnath, Yulia Krupskaya, Mahmoud Al-Hussein and Anton Kiriy**

Supporting Information

Charge-compensated n-doped π-conjugated polymers: toward both thermodynamic stability of n-doped states in water and high electron conductivity

Fabian Borrmann*†, Takuya Tsuda*†, Olga Guskova**†, Nataliya Kiriy†, Cedric Hoffmann,† David Neusser‡, Sabine Ludwigs‡, Uwe Lappan†, Frank Simon†, Martin Geisler†, Bipasha Debnath§, Yulia Krupskaya§, Mahmoud Al-Hussein‖, Anton Kiriy**†

†*Leibniz-Institut für Polymerforschung Dresden e.V., Hohe Strasse 6, 01069 Dresden, Germany*

‡*IPOC-Functional Polymers, Institute of Polymer Chemistry & Center for Integrated Quantum Science and Technology (IQST), University of Stuttgart, Pfaffenwaldring 55, 70569 Stuttgart, Germany*

§*Leibniz-Institut für Festkörper- und Werkstoffforschung Dresden, Helmholtzstrasse 20, 01069 Dresden, Germany*

‖*Physics Department and Hamdi Mango Center for Scientific Research, The University of Jordan, 11942 Amman, Jordan*

Synthesis

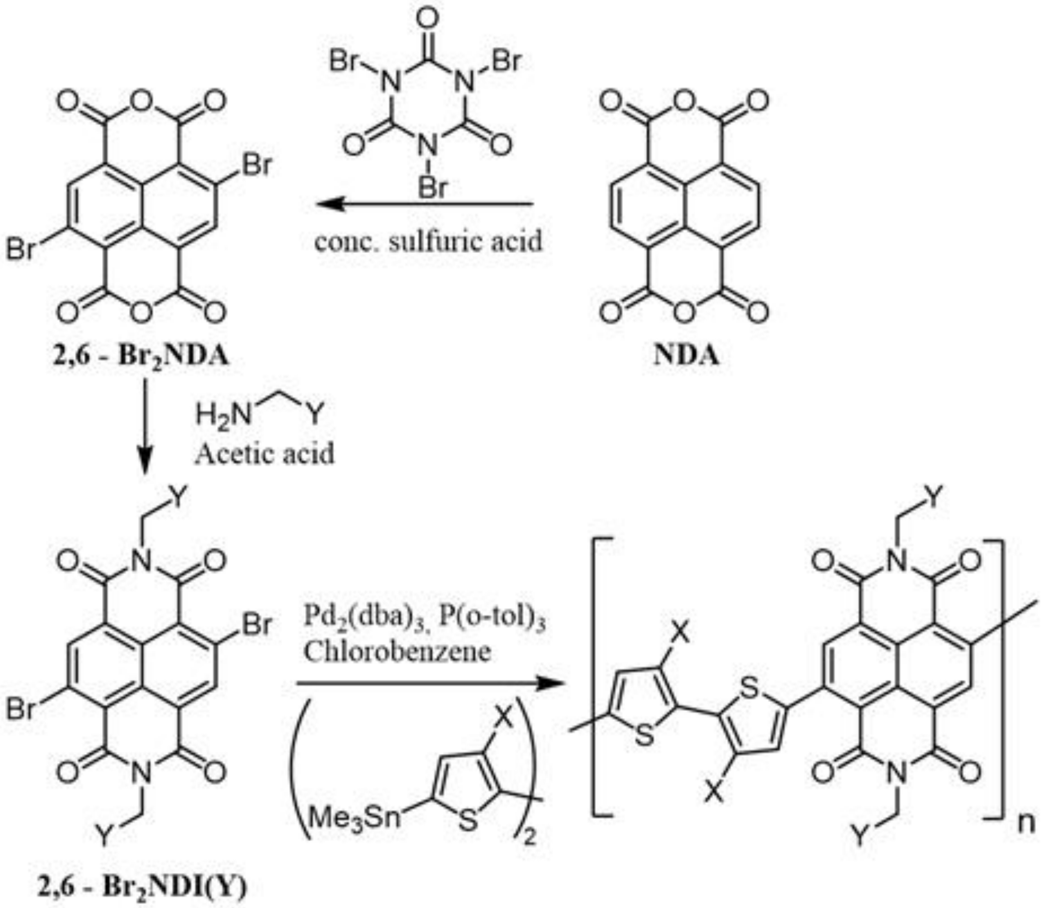

X=H: P(NDIC3AI-T2); X= F: P(NDIC3AI-T2F)

Figure S1. Synthesis of P(NDIC3AI-T2) and P(NDIC3AI-T2F).

Preparation of tribromoisocyanuric acid (TBCA)

To a stirred solution of cyanuric acid (16.66 mmol, 2.15 g), NaOH (50 mmol, 2 g) Na_2CO_3 (25 mmol, 2.65 g) and KBr (50 mmol, 5.95 g) in H_2O (300 mL) cooled in an ice bath was added dropwise a solution of potassium peroxymonosulfate (Oxone, 50 mmol, 30.73 g) in H_2O (250mL). During addition of the oxidant a white solid precipitate forming a dense suspension which is stirred for 20 h. The product is collected by vacuum filtration, washed with cold water and dried over P_2O_5. Yield is 89% (5.38 g).

Dibromination of naphathalene tetracarboxylic dianhydride (synthesis of 2,6-Br_2NDA)

To a stirred solution of naphthalene tetracarboxylic dianhydride (24.61 mmol, 6.6 g) in concentrated sulfuric acid (100 mL) TBCA (24.61 mmol, 9.01 g) was added in four portions over the course of 2 hours. After stirring in a sealed flask at room temperature for 4 days the reaction was poured in ice (900 mL) and allowed to warm up to room temperature. The resulting suspension was filtrated and the collected solid was washed with hot water and methanol (2x) leading to the raw product (7.12g, 55% Br_2NDA) which is stirred in acetone (500 mL) over night. The solid (4.6 g, 76% Br_2NDA) collected by vacuum filtration is recrystallized from DMF (52 mL) leading to the desired product Br_2NDA (2.7 g, >95% Br_2NDA) in 26% yield overall. ^{1}H-NMR (300 MHz, DMSO-d6), δ (ppm): 8.79 (s, 2H).

Alkylation of 2,6-dibromonaphthalene tetracarboxylic dianhydride (synthesis of 2,6-Br_2NDIC3A)

To Br_2NDA (2.16 mmol, 0.92 g) in glacial acetic acid (27 mL) *N*,*N*-dimethyl-1,3-propanediamine (6.16 mmol, 629.3 mg, 775 µL) was added slowly under inert conditions. The reaction mixture was heated up to 130 °C and stirred for 20 min under reflux. After cooling down to room temperature the mixture was poured into 130 mL crushed ice and neutralized with Na_2CO_3. The aqueous phase was extracted 5 times with 50 mL $CHCl_3$. The united organic phases were washed twice with water and brine. Then, they were dried with NaCl. $CHCl_3$ removal at the rotatory evaporator was stopped when a solid started to precipitate and the flask was placed in a cooler (-18°C) over night. The precipitate was filtrated and washed with Et_2O and dried to obtain Br_2NDI as yellow solid in 69% yield (880 mg). ^{1}H-NMR (300 MHz, $CDCl_3$), δ (ppm): 8.99 (s, 2H), 4.27 (t, 4H), 2.44 (t, 4H), 2.22 (s, 12H), 1.92 (m, 4H).

Stille polycondensation

These reactions took place in a 20 mL Schlenk flask which was pumped down to vacuum and baked out at 650°C for 10 min. After cooling down to room temperature it was backfilled three times with argon and all solids where added at argon counter flow which are: $Pd_2(dba)_3$ (0.02 eq, 3.08 mg), $P(oTol)_3$ (0.08 eq, 4.1 mg), Br_2NDI (1 eq, 0.168 mmol, 100 mg) and bithiophene (1 eq, 0.168 mmol, 82.76 mg of P(NDIC3AI-T2) and 88.82 mg of P(NDIC3AI-T2F). Once again, the flask is pumped down vacuum and backfilled with argon three times. 9 mL PhCl are added via syringe. Then, the reaction mixture is heated up and refluxed at 150 °C oil bath temperature for 1h. After cooling down to room temperature the reaction mixture is poured into Et_2O, filtrated and washed with the same solvent. After drying a black-blueish solid is obtained in quantitative yield which is the respective neutral polymer. Due to low solubility in usual solvents no NMR could be obtained.

Procedure for quaternization of polymers

The neutral polymer (1 eq, 100 mg) and an excess of MeI (>100eq, 1 mL) where stirred in 50 mL MeOH in an argon filled flask at room temperature overnight. After evaporating, the solid was dissolved in 10-15 mL DMSO and heated upon dissolution. This solution was dropped into 125mL toluene. The solid precipitate was filtrated, washed with $CHCl_3$ and dried to obtain the quaternized polymer as black-blueish solid in quantitative yield.

^{1}H-NMR (P(NDIC3AI-T2), 300 MHz, DMSO-d6), δ (ppm): 8.62 (s, 2H), 7.57 (d, 2H), 7.52 (d, 2h), 4.13 (t, 2H), 3.49 (t, 4H), 3.08 (s, 9H), 2.15 (m, 4H). Integrals of the signals in the spectrum somewhat deviate from a theoretically predicted values presumably due to aggregation of the polymers.

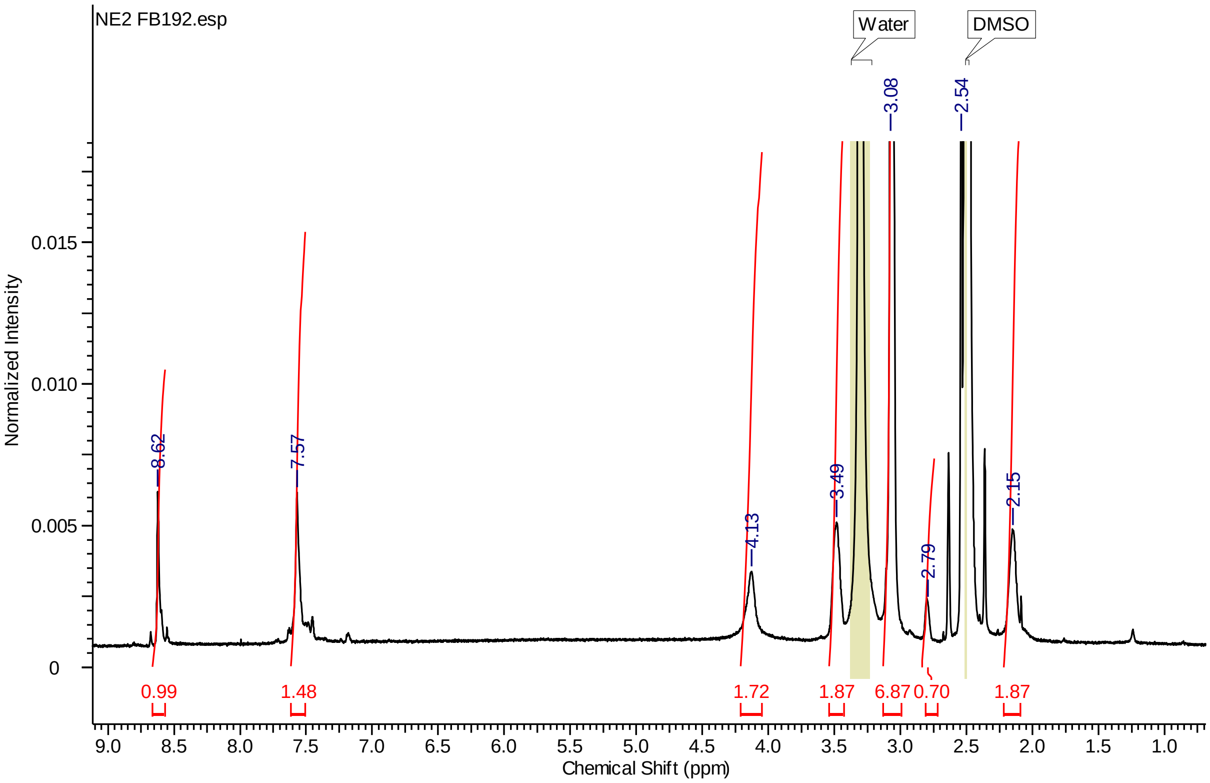

Figure S2. ^{1}H-NMR spectrum of (P(NDIC3AI-T2) taken in DMSO-d6.

^{1}H-NMR (P(NDIC3AI-T2F), 300 MHz, DMSO-d6), δ (ppm): 8.64 (s, 2H), 7.69 (d, 2H), 4.14 (t, 2H), 3.49 (t, 4H), 3.07 (s, 9H), 2.15 (m, 4H).

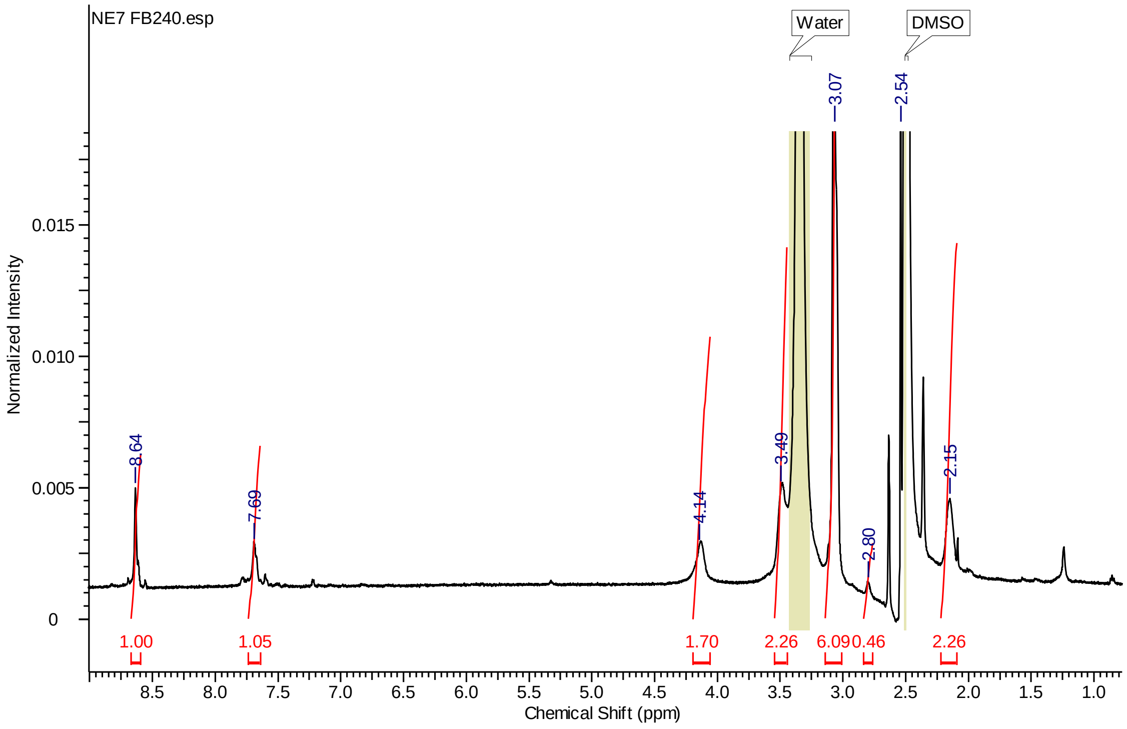

Figure S3. ^{1}H-NMR spectrum of (P(NDIC3AI-T2F) taken in DMSO-d6.

Dynamic Light Scattering (DLS)

Determination of the molecular weights of the ionic polymers by GPC due to the strong interactions with standard column materials does not seem possible. Instead, the dynamic light scattering (DLS) measurements were undertaken.

All samples were measured in a concentration of 0.2 mg/ml at 25 °C after 15 min ultrasonication dissolution. Sample 187 was measured in Chloroform. Samples 192b and 240 were measured in 2,2,2-Trifluoroethanol. The samples were measured in a 1 µl Quartz cuvette on a DynaPro® NanoStar® DLS by Wyatt Technology Corp. 15 acquisitions were averaged to one discrete measurement. Data recording and analysis was done applying Dynamics® Software by Wyatt Technology Corp., version 7.10.1.21. We note here, that the measurements in further dilution at 0.05 mg/mL were unsuccessful due to unstable auto-attenuation and high background noise.

Results of the regularization analysis

a) P(NDIOD-T2)

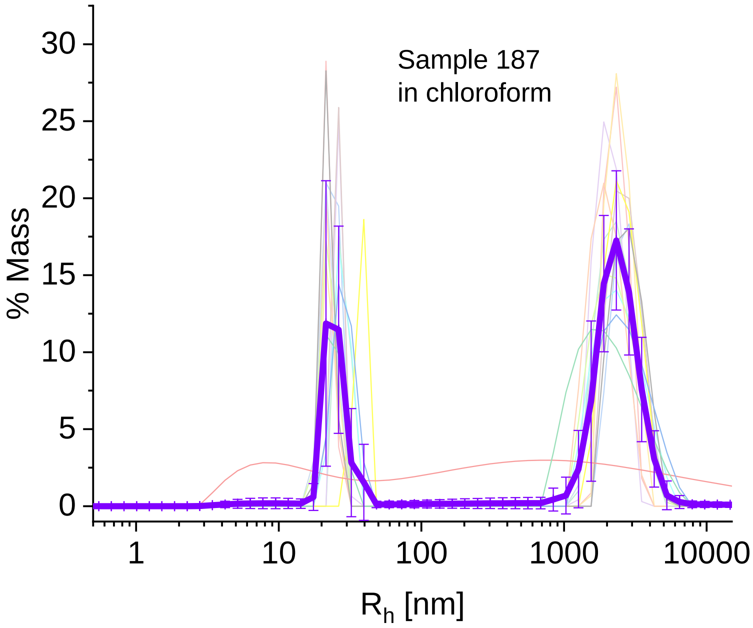

b) P(NDIC3AI-T2)

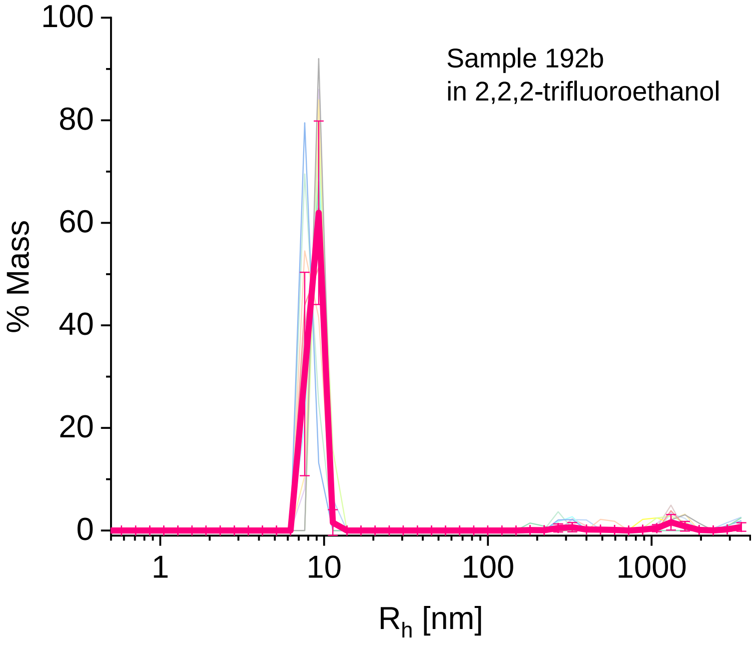

c) P(NDIC3AI-T2F)

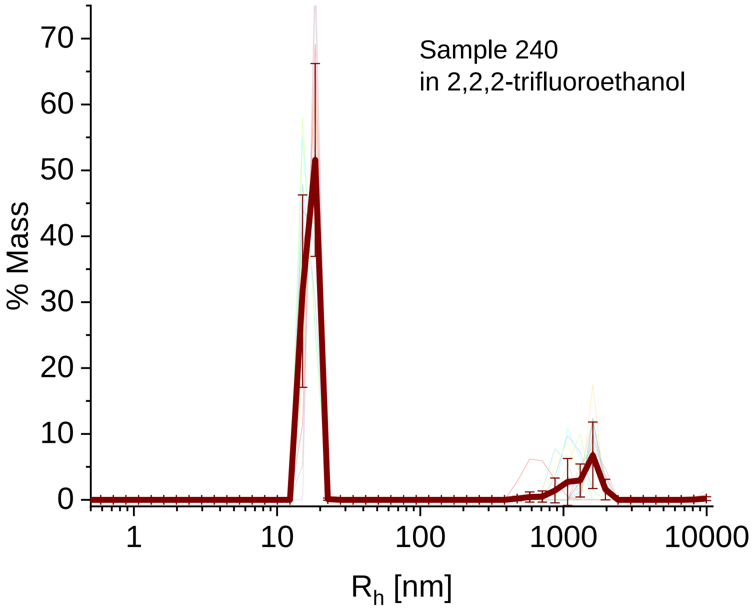

Figure S4. Individual curves of each discrete measurement and averaged curves (thicker lines): (a) P(NDIOD-T2); (b) P(NDIC3AI-T2); (c) P(NDIC3AI-T2F).

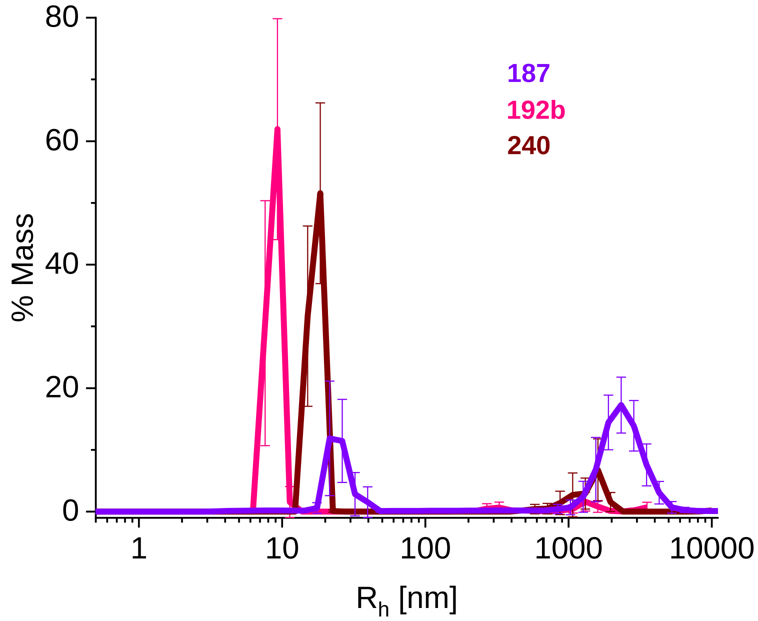

Figure S5. All average curves together: P(NDIOD-T2) (blue), P(NDIC3AI-T2) (red) and P(NDIC3AI-T2F) (braun).

Average values of the non-aggregated sample fraction:

Sample	D in cm²/s	Rh in nm
P(NDIOD-T2) (187)	(1.7 ± 0.2)E-7	25 ± 3
P(NDIC3AI-T2) (192b)	(1.42 ± 0.06)E-7	8.8 ± 0.4
P(NDIC3AI-T2F (240)	(7.3 ± 0.3)E-8	17.2 ± 0.6

Dynamic viscosities at 25 °C:

Chloroform: 0.542 mPa s

Reference:Chloroform Dynamic Viscosity

https://materials.springer.com/thermophysical/docs/vis_c47

Data from Dortmund Data Bank (DDB) – Thermophysical Properties

2,2,2-Trifluoroethanol: 1.7546 mPa s

Reference: Landolt-Börnstein - Group IV Physical Chemistry 25

(Supplement to IV/18) https://materials.springer.com/lb/docs/sm_lbs_978-3-540-75486-2_41

Degrees of polymerization and molar masses derived from the hydrodynamic sizes for P(NDIOD-T2), P(NDIC3AI-T2) and P(NDIC3AI-T2F)

According to previous simulations on the chain flexibility of naphthalenediimide – thiophene copolymers, a copolymer with two thiophene repeating units comprise a ratio of the gyration radius R_g to the root-mean-squared end-to-end-distance R_0 of $R_0/R_g = 3.04$,[1] which lies in between the ratio for flexible random coil polymers of 2.45 ($6^{0.5}$) and rigid rod architectures of 3.46 ($12^{0.5}$) [2]. This indicates a somewhat intrinsic semi-flexible polymer conformation for such type of co-polymer. Main-chain poly-(alkylthiophene) polymers exhibit also vastly a semiflexible chain conformation in solution [3,4]. The relation of the hydrodynamic radius to the gyration radius, so-coined rho-parameter $\rho = R_g/R_h$ for semiflexible polymers is about 2.0,[5,6] lying in between 1.5 for flexible linear chains in a thermodynamically good solvent and rigid rods at an upper limit of about 2.7 [7,8]. With the help of the ratio of the root-mean-squared end-to-end-distance to the maximum length of the polymer in fully "stretched" conformation $L/R_g = 4.34$, and $\rho = 2$, the hydrodynamic radius would resemble polymer sizes as given in **Table S1.**

Table S1. Calculated degree of polymerization (DP or N_{RU}) and molar mass based on a semiflexible chain conformation.

Sample	R_h in nm[#]	N_{RU} (or DP)	Molar mass [g/mol]
187	25 ± 3	39 ± 4	38000 ± 6000
192b	8.8 ± 0.4	14 ± 2	12200 ± 1300
240	17.2 ± 0.6	27 ± 3	25000 ± 2600

measured by DLS in Chloroform (187) or 2,2,2-trofluoroethanol (192b and 240)

Changes in the polymer chain flexibility due to solvent influence are respected by a rather large uncertainty in $\Delta\rho = \pm 0.2$.

Appendix:

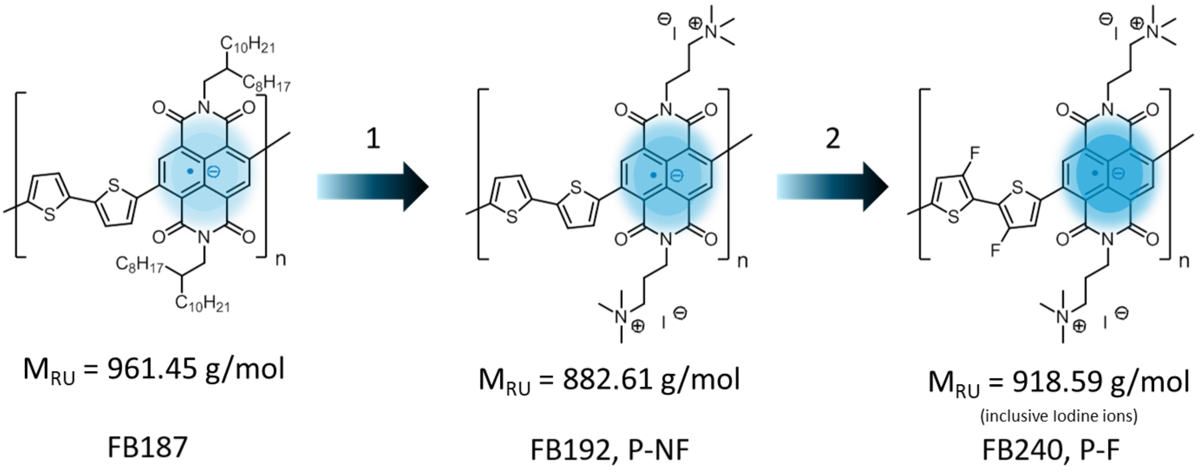

Length of a repeating unit:

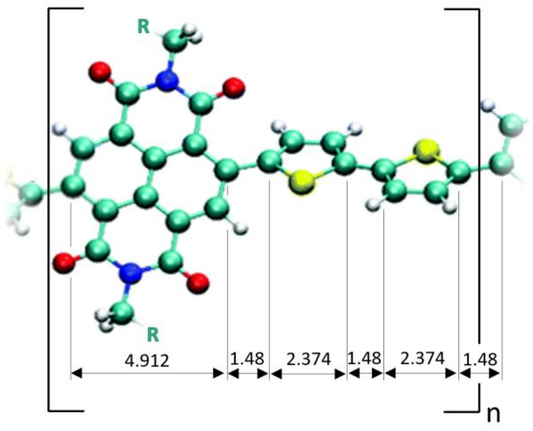

Σ = 14.1

lengths are given in Ångström (1 Å = 0.1 nm)

Figure taken from Ref. [1]

Geometry of thiophene taken from [9].

Example for 187 - Degree of polymerization (number of repeating units) N_{RU}:

measured R_h = 25 nm

1.) with $\rho = R_g / R_h = 2.0$ for semiflexible polymers → R_g = 12.5 nm
2.) with $L / R_g = 4.34$ according to SI of Ref. [1] (NDI-2T) → L = 55 nm
3.) with L_{RU} = 1.41 nm length per repeating unit (Σ = 14.1 Å from Figure above) → DP = N_{RU} = 39
4.) with M_{RU} = 961.45 g/mol Molar mass per RU → 37760 g/mol ≈ 38000 g/mol

summarized equation: $$N_{RU} = \frac{R_h \left[{}^{L}/_{R_g}\right] \rho}{L_{RU}}$$

Reasonable ± uncertainties:

$\Delta R_h = \pm 3$ nm

$\Delta\rho = \pm 0.2$

$\Delta L/R_g = \pm 0.05$

$\Delta L_{RU} = \pm 0.01$ nm

Root-mean-squared error (since uncertainties can partly compensate each other):

$$\Delta N_{RU} = \left[\frac{1}{n}\sum\left[\left|\frac{\partial x}{\partial N_{RU}}\right|\Delta x\right]^2\right]^{0.5} = \left[\frac{1}{4}\left(\left(\frac{\Delta R_h\, {}^{L}/_{R_g}\,\rho}{L_{RU}}\right)^2 + \left(\frac{R_h\,\Delta {}^{L}/_{R_g}\,\rho}{L_{RU}}\right)^2 + \left(\frac{R_h\, {}^{L}/_{R_g}\,\Delta\rho}{L_{RU}}\right)^2 + \left(\frac{R_h\, {}^{L}/_{R_g}\,\rho\Delta L_{RU}}{L_{RU}^2}\right)^2\right)\right]^{0.5}$$

Nominal exact values:

Sample	*R_h* from DLS	N_{RU}	Molar mass [g/mol]
187	25 ± 3	39.27 ± 6.03	37760 ± 5800
192b	8.8 ± 0.4	13.82 ± 1.50	12202 ± 1323
240	17.2 ± 0.6	27.02 ± 2.83	24821 ± 2596

Reasonably rounded values as given above in Table X:

Sample	*R_h* from DLS	N_{RU}	Molar mass [g/mol]
187	25 ± 3	39 ± 6	38000 ± 6000
192b	8.8 ± 0.4	14 ± 2	12200 ± 1300
240	17.2 ± 0.6	27 ± 3	25000 ± 2600

The rather large value of $\Delta\rho = \pm 0.2$ ($\rho = 1.8$ rather flexible coil; $\rho = 2.2$ rather stiff worm-like) already respects changes in flexibility due to varying solvent influence.

References:

[1] L. Ning, G. Han, Y. Yi, *J. Mater. Chem. C*, **2020**, 8, 16527—16532. DOI: 10.1039/D0TC03715J

[2] I. Teraoka, Polymer Solutions: An Introduction to Physical Properties. John Wiley & Sons, New York, 2002, p. 187.

[3] B. McCulloch, V. Ho, M. Hoarfrost, et al., *Macromolecules* **2013**, 46 (5), 1899–1907. DOI: 10.1021/ma302463d

[4] M. Nagai, J. Huang, T. Zhou, W. Huang, *J. Polym. Sci. Part B: Polym. Phys.*, **2017**, 55, 1273–1277. DOI: 10.1002/polb.24389

[5] T. Kume J. Araki, Y. Sakai, *et al. J. Phys.: Conf. Ser.* **2009**, 184, 012018. DOI: 10.1088/1742-6596/184/1/012018

[6] F. Dutertre, K.-T. Bang, E. Vereroudakis, et al. *Macromolecules* **2019**, 52 (9), 3342–3350. DOI: 10.1021/acs.macromol.9b00457

[7] Y. A. Skorik, V. A. Petrova, O. V. Okatova, et al. Macromol. Chem. Phys. 2016, 217, 1636–1644. DOI: 10.1002/macp.201600146

[8] W. Van de Sande, A. Persoons, J. Phys. Chem. **1985**, 89 (3), 404–406. DOI: 10.1021/j100249a007

[9] Kreher, Richard P.: Methoden der Organischen Chemie, (Houben-Weyl), E 6 a: Hetarene I; Thieme Verlag, Stuttgart, 1994, p. 186. DOI: 10.1055/b-0035-111248

Spectroelectrochemistry

Cyclic voltammograms were measured in an electrochemical three-electrode setup using standard electrolyte (0.1 M tetrabutylammonium hexafluorophosphate, $TBAPF_6$ in acetonitrile, MeCN) under argon atmosphere. Polymer films were deposited on gold substrates used as working electrodes against a platinum counter electrode. Since the experiments were carried out in organic electrolyte, a pseudo reference electrode made of a silver wire covered with a layer of AgCl was used. All potentials are referenced against the Fc^+/Fc redox couple added as standard after the measurement. LUMO energies were calculated by the following equation: E_{LUMO}=-5.1-[potential of the semiconductor vs ferrocene].

The experiments were conducted using a PGSTAT204 potentiostat from Metrohm (Filderstadt, Germany). Spectroelectrochemical experiments were measured on transparent ITO electrodes which were placed in the beam path of a diode array UV-vis spectrometer system from Zeiss (Oberkochen, Germany) equipped with a MCS621 vis II detector (Zeiss) and a CLH600 F halogen lamp (Zeiss).

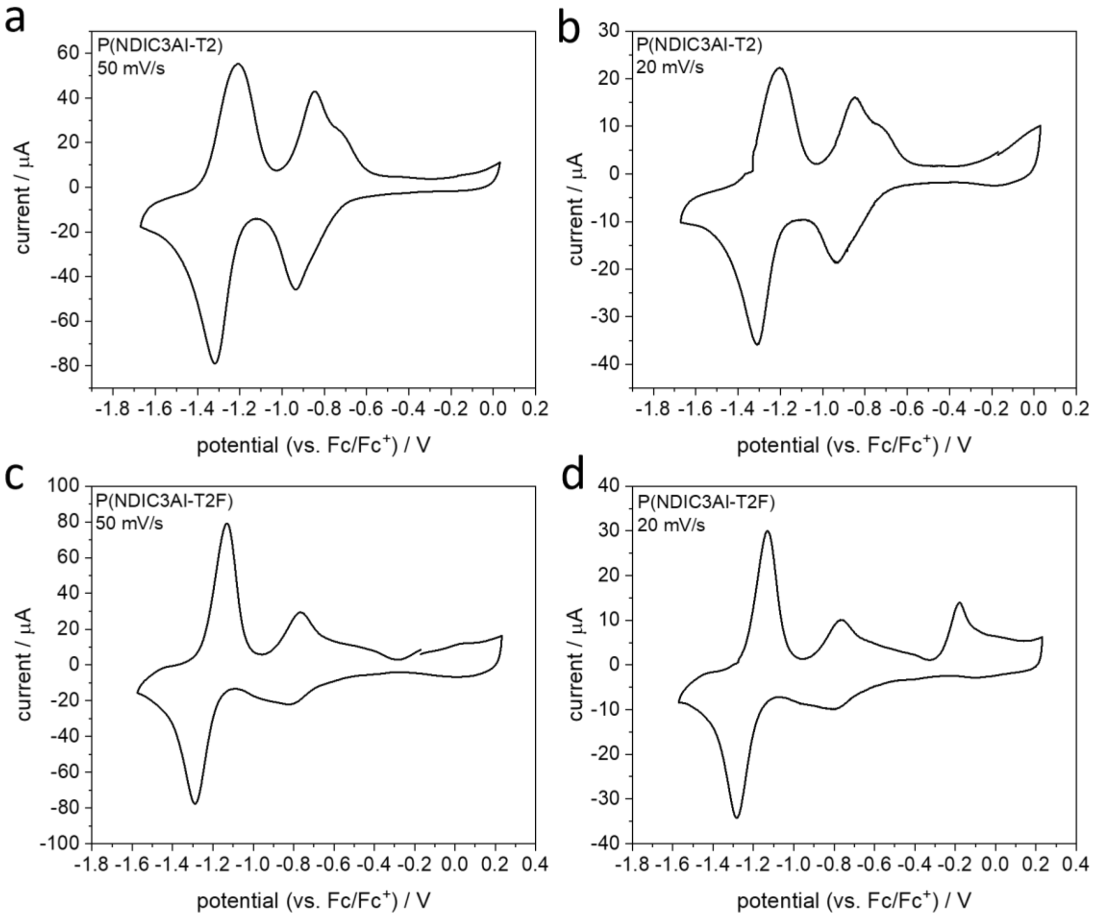

Figure S6. Cyclic voltammograms of P(NDIC3AI-T2) (a) and (b) and P(NDIC3AI-T2F) (c) and (d) measured on gold substrates in 0.1 M $TBAPF_6$ in MeCN. CV measurements were performed following the following procedure: Three cycles at 50 mV/s followed by three cycles at 20 mV/s. Graph (a) and (c) show the third cycle at 50 mV/s and graph (b) and (d) show the third cycle at 20 mV/s (sixth cycle in total). One can clearly see the increased charge trapping around 0 V in (b) and (d).

Preparation of the doped films

Polymer solutions. Usually polymers were prepared as 10 mg/mL solutions in trifluoroethanol (P(NDIC3AI-T2) and P(NDIC3AI-T2F)) and chloroform (P(NDIOD-T2)).

Pristine Polymer films. 50µL per each cm^2 of 10 mg/mL solutions were added on the substrate and spincoated at 700 rpm for 60s.

Dopant solutions. Cobaltocene was used as 1mg/mL solution in bis(2-methoxyethyl) ether (diglyme). A 1mg/mL sodium diphenylanthracenide solution was made by reacting sodium with diphenylanthracene in diglyme at 1:1 ratio. Oxalate was used as a 10.5 mg/mL solution in H_2O/MeOH (4:1).

Solid-state self-compensated doping. In a glove box the pristine polymer film is covered with cobaltocene (or sodium diphenylanthracenide) solution and left for 45 s. The solution is removed and the doped polymer surface is washed with pure diglyme which is removed as well. The polymer film is dried on a heat plate inside the glovebox at 100°C for 60 s.

Anion exchange doping. The pristine polymer film is mounted on a spin coater and covered by oxalate solution. After 45 s the solution is spun off at 1500 rpm (90 s). Pure water is added on top of the polymer film, left for 45 s and spun off at 1500 rpm (90 s).

Conductivity measurements

I-V characteristics were measured under ambient conditions at room temperature. The gold electrodes (5 nm Cr / 40 nm Au) with the geometry of 300 µm (length) × 4.5 mm (width) were formed by thermal evaporation through shadow masks on silicon substrates (n-type (100), 300 nm oxide layer). The films were spin-coated from trifluoroethanol solutions. The I-V measurements were conducted by a source measure unit of Keithley 2612B (Keithley Instruments, Solon, OH, USA) and a probe station by a simple two probe method.

Temperature-dependent conductivity

Au film contacts in a Hall bar geometry with channel length and width of 3 mm and 3.1 mm, respectively, were first deposited on a glass substrate. After that a 80 nm thin film of the investigated material was deposited on top of the contacts via spin coating. For the

measurements, the glass substrates with samples were mounted on a special chip carrier using Au wires and conducting silver epoxy. Temperature dependence of the conductivity was measured in a 4-probe configuration under vacuum using a He-flow cryostat from Oxford Instruments (Abingdon, UK) equipped with a special sample holder, a temperature controller from Lake Shore Cryotronics (Westerville, OH, USA) and a semiconductor parameter analyzer from Keysight Technologies (Santa Rosa, CA, USA).

FET measurements

Bottom contact-bottom gate FET measurements were performed similar to the temperature-dependent measurements, but the samples were prepared on a silicon wafer to realize the bottom gate.

GIWAXS

GIWAXS experiments were performed using GANESHA 300XL+ system (SAXSLAB ApS, Xenocs, Grenoble, France). The instrument is equipped with a Pilatus 300K detector, Cu X-ray source operated at 50 kV/0.6 mA (λ = 1.5408 Å), and a three-slit collimation system. The detector was moved in a vacuum chamber to a sample-to-detector distance of 110 mm and an incident angle of 0.2^{o}. The data were analyzed using GIXSGUI program.

EPR spectroscopy

Continuous wave (CW) EPR spectra were recorded on an EMX-plus spectrometer (Bruker Biospin, Billerica, MA, USA) operating at X-band, equipped with the high-sensitivity resonator ER 4119 HS-W1, and the variable temperature unit ER4141VT. Acquisition parameters were microwave power of 0.1 mW, modulation frequency of 100 kHz, modulation amplitude of 0.5 G, sweep width of 50 G, time constant of 10.24 ms, conversion time of 40.96

ms, 16 scans, and 1024 data points. For EPR, the polymer films were prepared on flexible FEP fluoropolymer sheets (25 µm).

Computational details

Following the standard procedure, the ground state geometries were optimized, the vibrational frequencies analysis of thiophene-flanked NDI-monomers with alkyl, amino-alkyl or positively charged ammonium side chains was performed with the Minnesota exchange-correlation energy functional M06-2X [1] and 6-31G(d,p) split-valence basis set for all the atoms except I, for which a double ζ valence basis set was applied [2]. The fluorinated analogues were modelled identically. Recently, the chosen functional and basis set was utilized in computational studies of NDI-containing polymers [3,4]. The frequency analysis confirmed that the optimized ground state structures represented true minima on the potential energy surface. All quantum-chemical calculations were carried out with the Gaussian 09 program package [5]. The molecular visualizations were prepared using Materials Studio [6]. When needed, the counterions (Cl^-, I^-) or cationic ions ($N(CH_3)_4^+$, Na^+) simulated explicitly have been added to the structures. The initial guess for the placement of a counterion was made as described elsewhere [7]. At the beginning of the calculations, the cations were located near the most negatively charged part of the molecule, i.e. near one of the oxygen atoms of NDI ring with ESP charge value ranging from -0.43 to -0.51 e, as obtained for propyl-substituted thiophene-flanked NDI-monomers. The partial atomic charges fitted to the electrostatic potential (ESP) were determined using the Merz-Singh-Kollman scheme [8]. The initial distance between Na cation and NDI oxygen corresponded to 3.5Å. If not stated otherwise, the adiabatic electron affinities are given by the difference between the energy of the neutral system at its most stable geometry, and of the anion, also at its most stable conformation.

To estimate a magnitude of different electron-donating effects introduced by alkyl substituents of different size, the NDI repeat unit with two methyl groups at the imide nitrogen atoms (NDIC1-T2) was also modelled. The reference (NDIC1-T2) with two methyl and (NDIC1-T5) two pentyl side groups have similar energies (-3.88 eV and -3.82 eV) indicating a small difference in inducting effects caused by a different size alkyl groups. Hence, we expect that the LUMO energy of the structure with $C_{20}H_{41}$ substituents should also be close to those values.

Data for NDIC3-T2F (LUMO=-4.06 eV, E^*=2.58 eV)

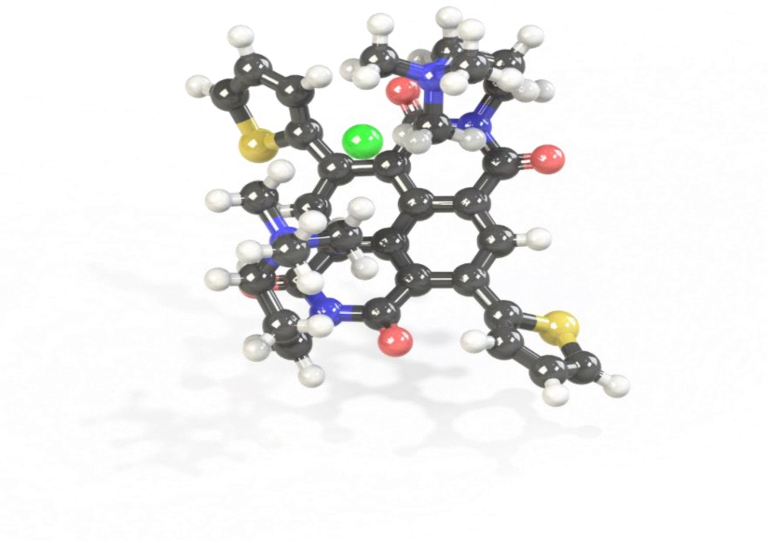

Figure S7. Snap-shot of the n-doped NDIC3A-T2(Cl^-) with one counter anion: a separation between two most distant carbons of two side chains is 4.0 Å. This snap-shot represents one of the possible geometries of such complexes, where two positively charged trimethylammonium side groups share among themselves one Cl^- anion, which is centered between them, and at the same time equally compensate the excess negative charge from the conjugated molecular core.

***References*:**

[1] Zhao, Y.; Truhlar, D.G. The M06 Suite of Density Functionals for Main Group Thermochemistry, Thermochemical Kinetics, Noncovalent Interactions, Excited States, and Transition Elements: Two New Functionals and Systematic Testing of Four M06-class Functionals and 12 other Functionals. *Theor. Chem. Acc.* **2008**, *120*, 215-241.

[2] Wadt, W.R.; Hay, P.J. Ab initio Effective Core Potentials for Molecular Calculations. Potentials for Main Group Elements Na to Bi. *J. Chem. Phys*. **1985**, *82*, 284-298.
[3] Liu, X.; Huang, C.; Wang, L.; Shen, W.; He, R.; Li, M. Theoretical Investigations on Naphthodithiophene Diimide-Based Copolymers as Acceptor for All-Polymer Solar Cell Applications. *ChemistrySelect* **2016**, *1*, 1662-1673.
[4] Wiberg, C.; Busch, M.; Evenäs, L.; Ahlberg, E. The Electrochemical Response of Core-Functionalized Naphthalene Diimides (NDI)–A Combined Computational and Experimental Investigation. *Electrochim. Acta* **2021**, *367*, 137480.
[5] Frisch, M.J.; Trucks, G.W.; Schlegel, H.B.; Scuseria, G.E.; Robb, M.A.; Cheeseman, J.R.; Scalmani, G.; Barone, V.; Mennucci, B.; Petersson, G.A.; et al. Gaussian 09, Revision D.01; Gaussian, Inc.: Wallingford, UK, 2009.
[6] BIOVIA Materials Studio. Dassault Systéms; Version 8; BIOVIA Materials Studio: San Diego, CA, USA, 2014.
[7] Montagna, M.; Guskova, O. Photosensitive Cationic Azobenzene Surfactants: Thermodynamics of Hydration and the Complex Formation with Poly(methacrylic acid). *Langmuir* **2018**, *34*, 311-321.
[8] Chandra, S.U.; Kollman, P.A. An Approach to Computing Electrostatic Charges for Molecules. *J. Comput. Chem*. **1984**, *5*, 129-145.

X-Ray Photoelectron spectroscopy (XPS)

All XPS studies were carried out by means of an Axis Ultra photoelectron spectrometer (Kratos Analytical, Manchester, UK). The spectrometer was equipped with a monochromatic Al Kα (h·ν = 1486.6 eV) X-ray source of 300 W at 15 kV. The kinetic energy of photoelectrons was determined with hemispheric analyzer set to pass energy of 160 eV for wide-scan spectra and 20 eV for high-resolution spectra. During all measurements, electrostatic charging of the sample was avoided by means of a low-energy electron source working in combination with a magnetic immersion lens. Later, all recorded peaks were shifted by the same value that was necessary to set the C 1s component peak showing the saturated hydrocarbons to a binding energy of 285.00 eV [1].

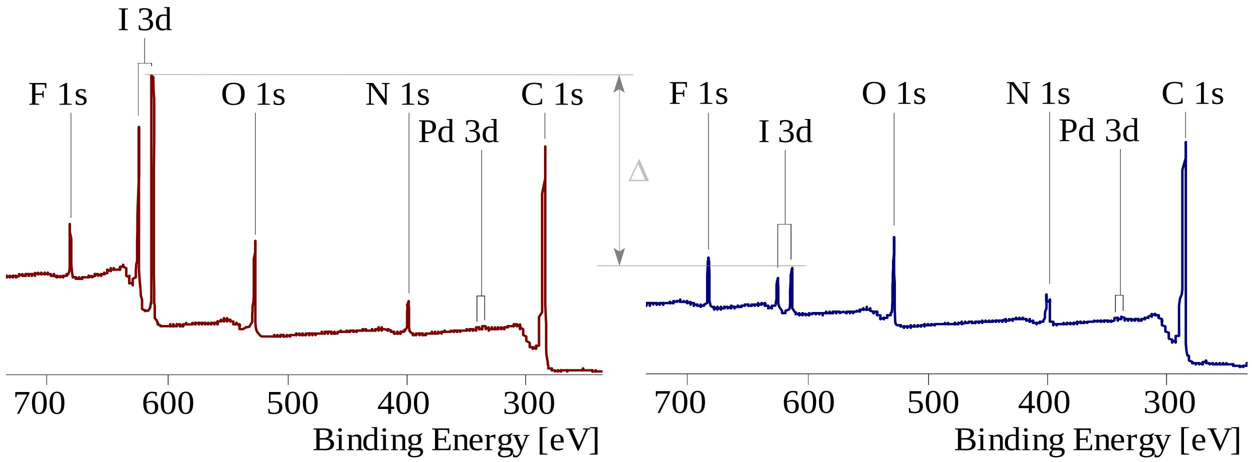

Figure S8. Wide-scan X-ray photoelectron spectra of pristine P(NDIC3AI-T2F) (left) and sodium oxalate-doped P(NDIC3AI-T2F) reveal a decrease (Δ) of iodide intensities by 82%.

Quantitative elemental compositions were determined from peak areas using experimentally determined sensitivity factors and the spectrometer transmission function. Spectrum background was subtracted according to Shirley [2]. The high-resolution spectra were deconvoluted by means of the Kratos spectra deconvolution software. Free parameters of component peaks were their binding energy (BE), height, full width at half maximum and the Gaussian-Lorentzian ratio. Figure S8 shows cut-outs from wide-scan photoelectron spectra recorded from the pristine and sodium oxalate-doped P(NDIC3AI-T2F) samples. As can be seen clearly, both of the two samples contain iodine, which was detected by the I 3d peaks. Due to the spin-orbit-interaction the I 3d peaks are composed of the I $3d_{5/2}$ (at 617.88 eV) and I $3d_{3/2}$ (at 629.36 eV) peaks. The coupling constant ($\Delta BE|_{I\ 3d} = |BE[3d_{5/2}] - BE[3d_{3/2}]|$, where BE is the binding energy) was found to be $\Delta BE|_{I\ 3d}$ = 11.48 eV. The intensities of the wide-scan spectra were normalized to the areas of the C 1s peaks (at 285.00 eV). The wide-scan spectrum of the pristine sample is characterized by an intense I 3d peak (atomic ratio [I]:[C] = 0.035). After replacing the iodine ions with oxalate ions, the relative iodine content was reduced to [I]:[C] = 0.006, which corresponds to a reduction of $\Delta \approx 82\%$.

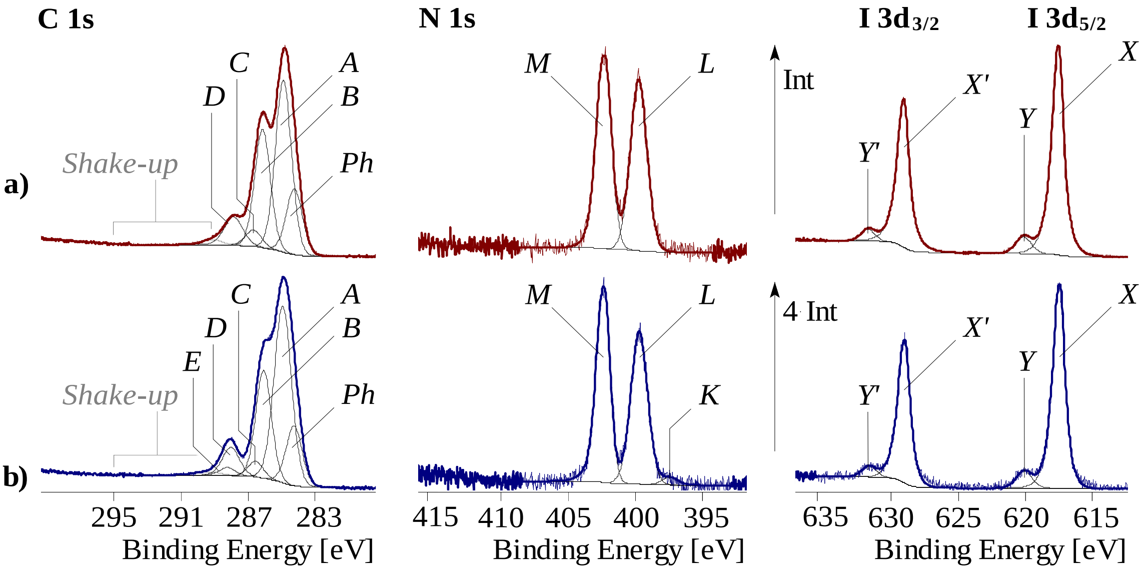

Figure S9. High-resolution C 1s, N 1s and I 3d X-ray photoelectron spectra of pristine P(NDIC3AI-T2F) (a) and sodium oxalate-doped P(NDIC3AI-T2F) (b). Compared to the intensity (Int) of the spectrum I 3d of sample P(NDIC3AI-T2F) (a), that of the I 3d spectrum of P(NDIC3AI-T2F) (b) sample was multiplied by four (4·Int).

The shapes of the high-resolution C 1s spectra confirm the chemical structure of the P(NDIC3AI-T2F) polymer (Figure S9). Most of the component peaks *A* at 285.00 eV resulted from saturated hydrocarbons ($^{A}\underline{C}_xH_y$) contaminating the film surface. The binding energy of the sulphur-bonded carbon atoms of the thiophene sequences ($^{A}\underline{C}$–S) is expected to be slightly less than 285 eV [3]. However, the low chemical shift of 0.1 eV did not allow the separation of a corresponding component peak, so that photoelectrons of the sulfur-bonded carbon atoms contributed also to component peak *A*. The photoelectrons escaped from the carbonyl carbon atoms of the imide groups (O=$^{D}\underline{C}$–N) were collected as component peaks *D* at 288.07 eV. The corresponding amine-sided carbon–nitrogen bonds and the other $^{B}\underline{C}$–N bonds of the quaternary ammonium groups ($^{B}\underline{C}$–N^{+}[–$^{B}\underline{C}$]$_3$) appear as component peaks *B* at 286.21 eV. The component peaks *B* also show the sp²-hybridized carbon atoms in the α-position of the carbonyl carbon atoms ($^{B}\underline{C}$–$^{D}\underline{C}$[=O]–N). All other photoelectrons from sp²-hybridized carbon

atoms were counted as component peaks *Ph* at 284.32 eV. The substitution of one hydrogen atom in the thiophene unit by fluorine resulted in C–F bonds, the photoelectrons of which were observed as component peaks *C* at 286.73 eV. The intensities found for these component peaks corresponded excellent at [B]:[CF] ≈ 7:1 to the stoichiometry of the P(NDIC3AI-T2F) polymer. Since the stoichiometric ratio of the polymer is $[B]:[D]|_{stoich}$ = 14:4, it was necessary to introduce an additional component peak *E* (at 288.28 eV) in the C 1s spectrum recorded from the sodium oxalate-doped P(NDIC3AI-T2F) sample for good spectral matching. This component peak shows the carbonyl carbon atoms of the oxalate ions ($^{-}OO^{E}\underline{C}–^{E}\underline{C}OO^{-}$) and their protonated species ($HOO^{E}\underline{C}–^{E}\underline{C}OO^{-}$ and $HOO^{E}\underline{C}–^{E}\underline{C}OOH$). Broad flat *shake-up* peaks (at binding energy values higher than 289 eV) resulted from photoelectrons escaped from polymer molecules in electronically excited states arising from $\pi \rightarrow \pi^*$ transitions.

Due to the co-presence of imide groups and quaternary ammonium groups in the P(NDIC3AI-T2F) polymer the N 1s spectra are characterized by clear splitting into the two component peaks *L* (at 399.9 eV) and *M* (at 402.3 eV). Due to the presence of cationic charges at the nitrogen atoms of the quaternary ammonium groups ($C–^{M}\underline{N}^{+}[–C]_3$) the component peaks *M* showed a high chemical shift of about 2 eV. The nitrogen atoms of the imide groups were represented by the component peaks *L* ($O{=}C–^{L}\underline{N}–C{=}O$). The intensity ratios found were [*L*]:[M] ≈ 1. The origin of the small component peak *K* found in the N 1s of the sodium oxalate-doped P(NDIC3AI-T2F) sample is not very clear. Its binding energy values of 397.68 eV is characteristic for inorganic nitrides and for nitrogen atoms interacting with the highly conjugated p-electron systems.

Our XPS spectra in the nitrogen region resemble spectra in Fig. 2 of the paper of Sunada et al. [4] recorded for a similar compound- perylene diimide having side groups with quaternized nitrogen groups. They claimed that if the charged group has OH- counterion, an easy elimination of methanol takes place leading to a tert-amine having in the N-region of the XPS signature a similar peak below 398eV.

It should be however noted that the binding energy values below 398eV (as found for component peak *K*, 397.68 eV) are very atypical for nitrogen atoms of amines. Independent of the number of alkyl rests bonded to the nitrogen atom (primary, secondary or tertiary amines) we expect to see binding energy values of between 399 and 400.5 eV ([1] appendix 4).

Binding energy values of ca. 398 eV (and smaller) were only found for inorganic nitrogen species and for organically bonded nitrogen atoms interacting with p-electron orbitals. So, we see such values for nitrogen in carboneous materials made from poly(acrylonitrile) [5] or in polydopamine films [6]. The peak K is therefore represents not an isolated amine but rather amine interacting (via the electron-transfer) with π-conjugated system.

Regarding the origin of the tert-amino group, it can appear in the polymer either due to 1) an incomplete quaternization or 2) due to the elimination. As the spectrum of the undoped sample does not contain the component K, we suggest that the second possibility is valid. Taking into account that the quaternized groups with I- counterion are not active in the elimination (according to [4], it proceeds on with OH-as counterion), and because undoped sample does not contain the component K, a plausible explanation is that some small amount of tert-amine was formed during the doping with oxalate. A water solution of sodium oxalate, which is a salt of a weak acid and a strong base, has a basic reaction, so that I- to OH- exchange is possible during the doping from water. The elimination and formation of the tert-amino group could happen during the sample drying process.

The exclusive presence of iodine ions should result in one component peak in the I $3d_{5/2}$ and I $3d_{3/2}$ spectra. As mentioned above, such intense component peaks *X* and *X'* were found at 617.88 eV [I $3d_{5/2}$ peak] and 629.36 eV [I $3d_{3/2}$ peak]. The binding energy values found are in the lower expected region for iodides [7].

Surprisingly the I 3d spectra showed clearly second maximums *Y* and *Y'* on the high-energy sides of the component peaks *X* and *X'*. According to their binding energy values of 620.62

eV [I $3d_{5/2}$ peak] and 631.93 eV [I $3d_{3/2}$ peak] these component peaks seem to be characteristic for iodomethane ($CH_3^{Y,Y'}I$) [8], which is a frequently used methylating agent.

References:

[1] Beamson, G.; Briggs, D.: High resolution of organic polymers. The Scienta ESCA 300 Database. J. Wiley & Sons, Chichester, New York, Brisbane, Toronto, Singapore, ISBN 0-471-93592-1 (1992), P. 26.

[2] D.A. Shirley: High-resolution X-ray photoemission spectrum of the valence bands of gold. Physical Review B: Condensed Matter and Materials Physics B5 (1972) 4709–4714.

[3] Stepien, L.; Roch, A.; Schlaier, S.; Dani, I.; Kiriy, A.; Simon, F.; von Lukowicz, M.; Leyens, C.: Investigation of the thermoelectric power factor of KOH-treated PEDOT:PSS dispersions for printing applications. Energy Harvesting and Systems 3 (2016) 101-112.

[4] Sunada, Y., Ishida, S.; Hirakawa, F.; Shiota, Y.; Yoshizawa, K.; Kanegawa, S., Sato, O.; Nagashima, H.; Iwamoto, T. Persistent four-coordinate iron-centered radical stabilized by π-donation, Chem. Sci. 7 (2016) 191-198.

[5] Zhu, S.; Göbel, M.; Formanek, P.; Simon, F.; Sommer, M.; Choudhury, S. Mask-painting symmetrical micro-supercapacitors based on scalable, poresize adjustable, N-doped hierarchical porous carbon. J. Mat. Chem. A 9 (2021) 14052-14063.

[6] Augustine, N.; Putzke, S.; Janke, A.; Drechsler, A.; Simon, F.; Zimmerer, C.: Dopamine-supported metallization of polyolefins - A contribution to an eco-friendly and efficient technological process. ACS Appl. Mat. Interf. 14 (2022) 5921-5931.

[7] https://srdata.nist.gov/xps/EngElmSrchQuery.aspx?EType=PE&CSOpt=Retri_ex_dat&Elm=i

[8] Zhou, X.L.; Solymosi, F.; Blass, P.M.; Cannon, K.C.; White J.M.: Interactions of methyl halides (Cl, Br and I) with Ag(111). Surface Science 219 [1-2] (1989) 294-316.
